# Exploitation of Olive (*Olea europaea* L.) Seed Proteins as Upgraded Source of Bioactive Peptides with Multifunctional Properties: Focus on Antioxidant and Dipeptidyl-Dipeptidase—IV Inhibitory Activities, and Glucagon-like Peptide 1 Improved Modulation

**DOI:** 10.3390/antiox11091730

**Published:** 2022-08-31

**Authors:** Martina Bartolomei, Anna Laura Capriotti, Yuchen Li, Carlotta Bollati, Jianqiang Li, Andrea Cerrato, Lorenzo Cecchi, Raffaele Pugliese, Maria Bellumori, Nadia Mulinacci, Aldo Laganà, Anna Arnoldi, Carmen Lammi

**Affiliations:** 1 Department of Pharmaceutical Sciences, University of Milan, 20133 Milan, Italy; 2 Department of Chemistry, Sapienza University of Rome, Piazzale Aldo Moro 5, 00185 Rome, Italy; 3 Longping Biotech Co., Ltd., Sanya 572000, China; 4 Department of Neuroscience, Psychology, Drug and Child Health, Pharmaceutical and Nutraceutical Section, University of Florence, 50019 Florence, Italy; 5 NeMO Lab, ASST Grande Ospedale Metropolitano Niguarda, 20162 Milan, Italy

**Keywords:** anti-diabetic activity, DPP-IV, GLP-1, *Olea europaea* L., antioxidant peptides

## Abstract

Agri-food industry wastes and by-products include highly valuable components that can upgraded, providing low-cost bioactives or used as an alternative protein source. In this context, by-products from olive production and olive oil extraction process, i.e., seeds, can be fostered. In particular, this work was aimed at extracting and characterizing proteins for *Olea europaea* L. seeds and at producing two protein hydrolysates using alcalase and papain, respectively. Peptidomic analysis were performed, allowing to determine both medium- and short-sized peptides and to identify their potential biological activities. Moreover, an extensive characterization of the antioxidant properties of *Olea europaea* L. seed hydrolysates was carried out both in vitro by 2,2-diphenyl-1-picrylhydrazyl (DPPH), by ferric reducing antioxidant power (FRAP), and by 2,2′-Azino-bis(3-ethylbenzothiazoline-6-sulfonic acid) diammonium salt (ABTS) assays, respectively, and at cellular level by measuring the ability of these hydrolysates to significant reduce the H_2_O_2_-induced reactive oxygen species (ROS) and lipid peroxidation levels in human intestinal Caco-2 cells. The results of the both hydrolysates showed significant antioxidant properties by reducing the free radical scavenging activities up to 65.0 ± 0.1% for the sample hydrolyzed with alcalase and up to 75.7 ± 0.4% for the papain hydrolysates tested at 5 mg/mL, respectively. Moreover, similar values were obtained by the ABTS assays, whereas the FRAP increased up to 13,025.0 ± 241.5% for the alcalase hydrolysates and up to 12,462.5 ± 311.9% for the papain hydrolysates, both tested at 1 mg/mL. According to the in vitro results, both papain and alcalase hydrolysates restore the cellular ROS levels up 130.4 ± 4.24% and 128.5 ± 3.60%, respectively, at 0.1 mg/mL and reduce the lipid peroxidation levels up to 109.2 ± 7.95% and 73.0 ± 7.64%, respectively, at 1.0 mg/mL. In addition, results underlined that the same hydrolysates reduced the activity of dipeptidyl peptidase-IV (DPP-IV) in vitro and at cellular levels up to 42.9 ± 6.5% and 38.7 ± 7.2% at 5.0 mg/mL for alcalase and papain hydrolysates, respectively. Interestingly, they stimulate the release and stability of glucagon-like peptide 1 (GLP-1) hormone through an increase of its levels up to 660.7 ± 21.9 pM and 613.4 ± 39.1 pM for alcalase and papain hydrolysates, respectively. Based on these results, olive seed hydrolysates may represent new ingredients with antioxidant and anti-diabetic properties for the development of nutraceuticals and functional foods for the prevention of metabolic syndrome onset.

## 1. Introduction

The use of food-derived bioactive peptides for the development of functional foods and/or nutraceuticals it is becoming an attractive practice, and currently, products containing peptides with health-promoting effects are accessible on the market [1]. Over the last ten years, a considerable number of scientific investigations have highlighted the beneficial effects of protein hydrolysates and peptides recovered from a wide range of food by-products, including antimicrobic, antihypertensive, and especially antioxidant and antidiabetic [2,3,4,5,6,7,8,9]. By-products are secondary products obtained from primary agro-food production processes and traditionally disposed of in landfills, with consequent air and water pollution and soil contamination. Food waste, by-products, and side-streams from several agricultural and food industries represent an attractive source of potentially functional or bioactive compounds, which can easily find application in the nutraceutical and pharmaceutical fields as an economic source [10]. In this context, the literature reports the presence of several food-origin peptides with antioxidant properties and hypoglycemic activity, such as dipeptidyl peptidase-IV (DPP-IV) inhibitors [11,12,13]. Indeed, these two biological activities are among the most studied; however, the combination of their biological effects could be further investigated. Generally, a high oxidative state is found in individuals with diabetes mellitus (DM), where the main molecular mechanisms involved are related to glucose and lipid metabolism. Rudich and Maddux observed a decrease in glucose uptake, in oxidative stress condition, and in muscle and fat cells and the reduction of the quantity and quality of insulin secreted by pancreatic β-cells [14,15]. Additionally, the over-production of reactive oxygen species (ROS) in β-cells lead to cell damage and activation of pathways involved in DM complications, i.e., neuropathy, endothelial dysfunction, nephropathy, and retinopathy [16,17]. A novel approach to control diabetes is based on incretin hormone glucagon-like peptide 1 (GLP-1), which normalizes blood glucose levels and reduces postprandial glycemia by stimulating insulin secretion [18]. Since DPP-IV is a serine protease responsible for the rapid degradation of GLP-1 in blood plasma [19], the use of inhibitors of this enzyme could prolong the half-life of GLP-1 by increasing its levels.

Considering the beneficial effects of bioactive peptides on human health, various antioxidant and/or hypoglycemic peptides were obtained from the recovery of food by-products [20,21,22] and bioactive molecules, such as lignans, tocopherols, flavonoids, secoiridoids, and especially hydroxytyrosol, and have already been obtained from waste and by-products of olive production [23,24,25,26,27], which generates 16 million tons of green and black table olives and 2.7 million tons of oil, according to FAOSTAT (Food and Agriculture Organization Corporate Statistical Database), and 95% is produced mainly in Spain and Italy.

In this scenario, this study focuses on fostering the olive stones or seeds (OS), a lignocellulosic, low-moisture by-product obtained after separation through horizontal centrifugation of the crushed seeds, peels, and pulp. OSs are good source of dietary fiber but also lipids and proteins rich in essential amino acids, especially valine and arginine [28]. Therefore, the first objective of this work was the physicochemical and conformational characterization of the total proteins extracted from three different cultivars of *Olea europaea* L., i.e., *Leccino*, *Moraiolo*, and *Frantoio*, by a combination of different techniques. Hence, total proteins were hydrolyzed using two food-grade enzymes, i.e., alcalase and papain, in the optimal conditions to obtain potential bioactive peptides.

The second objective of the work was to evaluate the potential biological effects of the olive stone hydrolysates, specifically the antioxidant effects of the extracted peptides, using in vitro and cellular assays. Considering that the modulation of the DPP-IV enzyme can occur in the presence of oxidative stress [29], in parallel, the antidiabetic activity was evaluated by investigating the in vitro and cellular inhibition of the DPP-IV enzyme. In addition, the modulation of incretin hormones GLP-1 levels was assessed through an innovative assay based on a co-culture system. The aim was to simulate the intestinal barrier in which the GLP-1 hormone is physiologically produced by endocrine cells (L cells of the intestinal mucosa) through the cleavage of proglucagon. Here, murine intestinal secretin tumor cell line (STC-1) was used as a model in order to achieve GLP-1 production; the third objective of the work was to investigate.

## 2. Materials and Methods

### 2.1. Chemical and Samples

All chemicals and reagents were commercially available, and more details are reported in the Supplementary Materials.

### 2.2. Protein Extraction from Olive Seed

Olive seed samples were obtained from the fruits of three cultivars, namely *Leccino*, *Frantoio*, and *Moraiolo*, harvested in Tuscany (soc. agr. Buonamici, Fiesole). Each sample was obtained mixing the fruits collected at different ripening degrees from September to November. The pulp was separated from the kernel by a spatula, the nuts were manually broken with a hammer, and the whole seeds were removed. From 100 g of dried olives were obtained the following mean yields: *Leccino* 4.0%, *Frantoio* 3.2%, and *Moraiolo* 3.0%. Olive seeds were grounded with a domestic mill. Olive seed powder was defatted with hexane for 1 h (ratio 1:20 *w*/*v*) under magnetic stirring. After drying, olive seeds were grounded with a domestic mill. Olive seed powder was defatted with hexane for 1 h (ratio 1:20 *w*/*v*) under magnetic stirring. After drying, the defatted powder was subjected to protein extraction. In detail, 1.5 g of defatted powder were mixed with 30 mL of extracting solution containing UREA 6 M, 0.1 M Tris-HCl (pH 8), 0.5 M NaCl, 0.5% SDS, and 0.1% DTT. More details are reported in the Supplementary Materials.

### 2.3. Protein Solubility (PS), Water-Binding Capacity (WBC), and Oil-Binding Capacity (OBC)

PS and OBC were determined according to a method described previously [30] with slight alterations. WBC was assessed as previously described [31]. More details are reported in the Supplementary Materials.

### 2.4. Free-Sulfhydryl Group Determination

Ellman’s reagent (DTNB) is a compound used for quantifying free-sulfhydryl groups in solution, observing the production of a yellow-colored product when it reacts with sulfhydryl groups. More details are reported in the Supplementary Materials.

### 2.5. Intrinsic Fluorescence Spectroscopy

The intrinsic fluorescence spectrum of each sample was obtained using a fluorescence spectrophotometer (Synergy H1, Biotek, Bad Friedrichshall, Germany). More details are reported in the Supplementary Materials.

### 2.6. Raman Spectroscopy

Raman analysis was conducted using Progeny™ spectrophotometer (Rigaku Corporation, Akishima, Japan) with a 1064 nm laser source and selectable laser output set at 490 mW. The spectral range was 200–2500 cm^−1^ with transmission-type volume phase grating. The spectral resolution was 15–18 cm^−1^, and the detector is a thermoelectrically cooled indium gallium arsenide (InGaAs). The samples were analyzed with 60 cumulative scans with optimized laser power, aperture size, and duration (7 s) per exposure in order to achieve the best signal-to-noise ratio. All of the obtained spectra were reported using Origin™ 8 software (OriginLab Corporation, Northampton, MA, USA).

### 2.7. Olive Seed Protein Hydrolysis for Releasing Bioactive peptides

The enzymatic hydrolysis of olive seed proteins was performed using alcalase and papain enzymes using optimal hydrolysis conditions (Table 1). All recovered peptides were lyophilized and stored at −80 °C until use. The DH (%) for each hydrolysate was identified by the o-phthaldialdehyde (OPA) method as previously described [32]. More details are reported in the Supplementary Materials.

### 2.8. Short Peptide Purification and Analysis by High-Performance Liquid Chromatography–High-Resolution Mass Spectrometry

Before analysis, short-sized peptides were purified from longer peptides and other macromolecules using a solid-phase extraction cartridge packed with 500 mg of carbograph 4 with a procedure that was optimized in a previous study [33]. The purified samples were then subject to HPLC-HRMS analysis using a Vanquish binary pump H (Thermo Fisher Scientific, Milan, Italy) coupled through a heated electrospray (ESI) source to a hybrid quadrupole–Orbitrap mass spectrometer Q Exactive (Thermo Fisher Scientific, Milan, Italy). More details are reported in the Supplementary Materials.

### 2.9. Profile of Potential Biological Activities and Peptide Ranking

The open-access tool PeptideRanker, a web-based tool used to predict the eventuality of biological activity of peptide sequences, was used to forecast the potential bioactivities of olive seed peptides [30]. Using N-to-1 neural network probability, PeptideRanker provides peptide scores in the range of 0–The peptides with a score higher than 0.6 were considered as potentially “bioactive”. Subsequently, the lists of best-ranked peptides were submitted to the web-available database BIOPEP.

### 2.10. Cell Culture

Caco-2 cells, kindly obtained from INSERM (Paris, France), and STC-1, commercialy available from ATCC (HB-8065, ATCC from LGC Standards, Milan, Italy), were routinely sub-cultured following a previously optimized protocol [34]. More details are reported in the Supplementary Materials.

### 2.11. 3-(4,5-Dimethylthiazol-2-yl)-2,5-Diphenyltetrazolium Bromide (MTT) Assay

A total of 3 × 10^4^ cells/well were seeded in 96-well plates and treated with different concentrations of hydrolysates and/or vehicle (H_2_O) in complete growth medium for 48 h at 37 °C under a 5% CO_2_ atmosphere. Experiments were performed by a standard method with slight modifications [35], and more details are provided in Supplementary Materials.

### 2.12. Antioxidant Activity of Olive Seed Hydrolysates

#### 2.12.1. Diphenyl-2-Picrylhydrazyl Radical (DPPH) Assay

The DPPH assay was performed by a standard method with a slight modification. The experimental method is detailed in the Supplementary Materials.

#### 2.12.2. 2,2′-Azino-Bis(3-Ethylbenzothiazoline-6-Sulfonic Acid) Diammonium Salt Assay

The Trolox equivalent antioxidant capacity (TEAC) assay is based on the reduction of the 2,2-Azino-bis-(3-ethylbenzothiazoline-6-sulfonic acid) (ABTS) radical induced by antioxidants. The experimental method is detailed in the Supplementary Materials.

#### 2.12.3. FRAP Assay

The FRAP assay evaluates the ability of a sample to reduce ferric ion (Fe^3+^) into ferrous ion (Fe^2+^). The experimental method is detailed in the Supplementary Materials.

#### 2.12.4. Fluorometric Intracellular ROS Assay

For cells preparation, 3 × 10^4^ Caco-2 cells/well were seeded on a black 96-well plate overnight in growth medium. The experimental method is detailed in the Supplementary Materials.

#### 2.12.5. Lipid Peroxidation (MDA) Assay

Furthermore, 2.5 × 10^5^ Caco-2 cells/well were seeded in a 24 well plate, and the following day, they were treated the OA and OP hydrolysates for 24 h at 37 °C under 5% CO_2_ atmosphere. The experimental method is detailed in the Supplementary Materials.

### 2.13. Antidiabetic Activity of Olive Seed Hydrolysates

#### 2.13.1. In Vitro Measurement of the DPP-IV Inhibitory Activity

The experiments were carried out in triplicate in a half-volume 96-well solid plate (white) using conditions previously optimized [36]. More details are reported in the Supplementary Materials.

#### 2.13.2. Evaluation of the Inhibitory Effect of Olive Seed Hydrolysates on Cellular DPP-IV Activity

A total of 3 × 10^4^ cells/well were seeded in black 96-well plates with a clear bottom. The second day after seeding, the spent medium was discarded, and cells were treated with 1.5 and 5.0 mg/mL of olive seed hydrolysates for 1 h at 37 °C. Experiments were carried out following previously optimized conditions [37]. More details are available in the Supplementary Materials.

#### 2.13.3. Evaluation of the GLP-1 Stability and Secretion at Cellular Level

STC-1 GLP-1 secretion was measured by an active GLP-1 ELISA kit (catalog no. EGLP-35K; Millipore, Watford, UK) read on a a Synergy H1 fluorescence microplate reader from BioTek, Milan Italy. More details are available in the Supplementary Materials.

### 2.14. Statically Analysis

All measurements were performed in triplicate, and results were expressed as the mean ± standard deviation (s.d.), where *p*-values < 0.05 were considered to be significant. All the data sets were checked for normal distribution by D’Agostino and Pearson test. Since they are all normally disturbed with *p*-values < 0.05, we proceeded with statistical analyses. Statistical analyses were performed by one- and two-way ANOVA followed by Dunnett’s and Tukey’s post-test (Graphpad Prism 9, GraphPad Software, La Jolla, CA, USA).

## 3. Results

### 3.1. Extraction of Olive Seed Proteins

The *Olea europaea* L. seed proteome is predominantly composed of seed storage proteins (SSPs), which are fundamental molecules for the germination phase and rich in carbon and nitrogen [23,24]. In most of the already available studies, olive seed proteins are extracted by using Tris-HCl buffer in reducing conditions [38,39,40]. In order to obtain both a good extraction yield and a favorable variety of proteins, in this study, different extraction buffers and methods were tested, and the best one was selected for all the further analysis. In order to optimize the extraction method of olive seed protein, three main buffers were investigated (Table 2).

**Figure 1:**
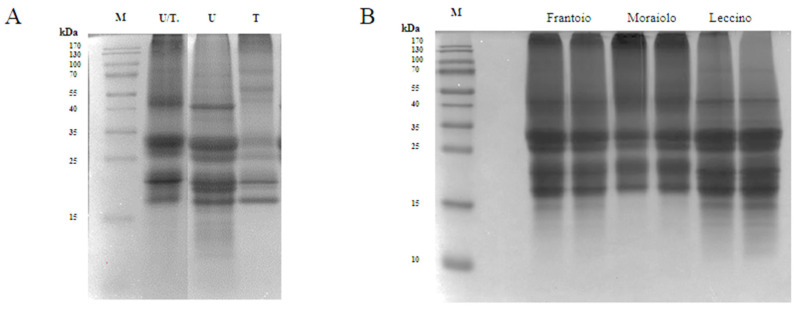
Proteins (50 μg) were loaded in each lane, and protein size markers are indicated in kilodaltons (kDa). (**A**) SDS-PAGE of olive seed proteins extracted with different buffers U/T: UREA 6 M, 0.1 M Tris-HCl (pH 8), 0.5 M NaCl, 0.5% SDS, 0.1% DTT; U: UREA 8 M, 1% CHAPS, 0.1% DTT, T: 0.1 M Tris (pH 7.5), 0.5 M NaCl, 0.5% SDS, 0.1% DTT. (**B**) SDS-PAGE of different cultivars of olive seed proteins extracted with the final buffer. For each cultivar were performed two different extractions.

The use of UREA 8 M buffer (U) allowed to obtain an excellent extraction yield of 9.2 mg/mL. Three intense bands were detected in the U lane of the SDS-PAGE at 41 kDa corresponding to the large precursor of the 11S proteins (Pro2), at 30.5 kDa corresponding to the 11S globulin subunit β-like protein, and at ~18 kDa corresponding to the 11S globulin seed storage protein 2 (Figure 1A) [12]. Tris-HCl buffer (T) showed the worst performance with a yield of 1.9 mg/mL. As underlined in the SDS-PAGE, the 40 kDa band was missing, and most of the other bands were detected in the 20–18 kDa range, which could be attributed to SSPs from Solea I precursor. Even though U buffer was the best compared to T buffer, it is known that urea can interfere with the activity of different enzymes, such as the proteases, therefore causing a problem during the hydrolysis process [11]. Therefore, the buffer containing both urea at a lower concentration of 6 M and Tris-HCl was selected as the best condition for the extraction process obtaining a yield of 5.3 mg/mL. As can be seen from the SDS-PAGE, in the U/T lane, the most intense bands were obtained in the range from 25 to 30 kDa, which could match the SSPs from the Solea II precursor, and in the range from 20 to 18 kDa (Figure 1A) [13]. To further reduce the urea concentration, the extracted proteins were subjected to dialysis against 1 M Tris-HCl (pH 8). Ultrasound pretreatment was also applied for improving extraction yield, but this strategy did not bring any significant advantage, and therefore it was excluded. Indeed, the extraction yield obtained using ultrasound/U buffer was 10.7 mg/mL compared to 9.2 mg/mL obtained using U buffer alone. Comparing the extraction yields obtained using T buffer with and without ultrasound pre-treatment, no significant differences were observed.

As we can see in the SDS-PAGE (Figure 1B), where we loaded the proteins that were extracted from the three different cultivars, (*Frantoio*, *Moraiolo*, and *Leccino)* using the U/T buffer, we observed significant bands at 30.5 kDa and at 20–18 kDa. In *Frantoio* and *Moraiolo*, stronger bands at 41 kDa were observed. Below 15 kDa, on the other hand, we observed no bands only in the *Moraiolo* sample.

### 3.2. Structural Properties Characterization: Raman Spectroscopy, Free-Sulfhydryl Group (SH) Content, and Intrinsic Fluorescence (IF)

**Figure 2:**
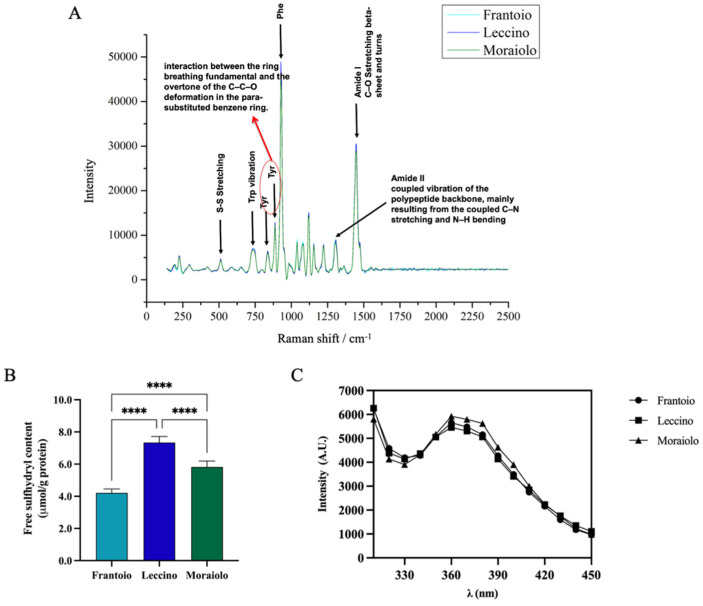
(**A**) Secondary structure analysis by Raman spectroscopy (125–2500 cm^−1^ region). Tertiary structure analysis. (**B**) Free-SH group determination. (**C**) Intrinsic fluorescence signal detection. Statistical analysis was performed by one-way ANOVA followed by Tukey’s post hoc test (****) *p* < 0. The data are represented as the means ± s.d. of three independent experiments.

The secondary structure of *Frantoio*, *Leccino*, and *Moraiolo* proteins was assessed by Raman spectroscopy (Figure 2A). Raman spectra of all samples revealed similar vibrational peaks. In the amide I region, all samples showed vibrational peak found in β-sheet structures and turns ascribable to the C–O stretching. In the amide II region, a narrow peak is associated with the coupled vibration of the polypeptide backbone, mainly resulting from the coupled C–N stretching and N–H bending. A narrow band at 1000 cm^−1^ is associated with Phe vibration. Moreover, the bands observed at 500 and 750 cm^−1^ are assigned to S–S stretching and Trp vibration, respectively. Further bands at approximately 831 cm^−1^ and 850 cm^−1^ are ascribable to interaction between the ring breathing fundamental and the overtone of the C–C–O deformation in the para-substituted benzene ring of Tyr.

Sulfhydryl (SH) groups and disulfide bonds (S-S) have a significant influence on functional properties of proteins, such as foaming and emulsifying abilities [41]. The evaluation of the content of free-SH groups located on the surface of olive seed protein was used to evaluate if there were differences between the different olive cultivars and to confirm the denatured state of the proteins. Figure 2B shows significant difference in free-SH content between cultivars. In details, the free-SH content was 4.2 ± 0.25% μmol/g, 7.3 ± 0.37% μmol/g, and 5.8 ± 0.37% for *Frantoio, Leccino*, and *Moraiolo* olive seed total protein extract (OTPE), respectively (****, *p* < 0.0001). These findings surely indicate that the protein structure in *Frantoio* presents a reduced exposure of the hydrophobic amino acids containing thiol groups, likely due to the formation of intermolecular disulfide bonds (S-S), whereas the *Leccino* sample shows higher free-thiol group content than the *Moraiolo* and *Frantoio* samples, respectively, indicating that in this sample, hydrophobic amino acids such as cysteine (Cys) and methionine (Met) are exposed on the surface of the protein structure. More information about the 3D protein structure was obtained by applying intrinsic fluorescence spectroscopy, a technique that can be used to monitor the fluoresce due to the presence of specific amino acids in proteins, i.e., phenylalanine (Phe), tyrosine (Tyr), and tryptophan (Trp). Figure 2C shows two main peaks at 300–310 nm and 360–370 nm, which represent Tyr in a hydrophilic environment, confirming the denatured state of the proteins in agreement with the results obtained from the characterization of the free-thiol groups. All the extracted proteins show comparable IF values.

### 3.3. Protein Solubility (PS), Water-Binding Capacity (WBC), and Oil-Biding Capacity (OBC) of Extracted Proteins

**Figure 3:**
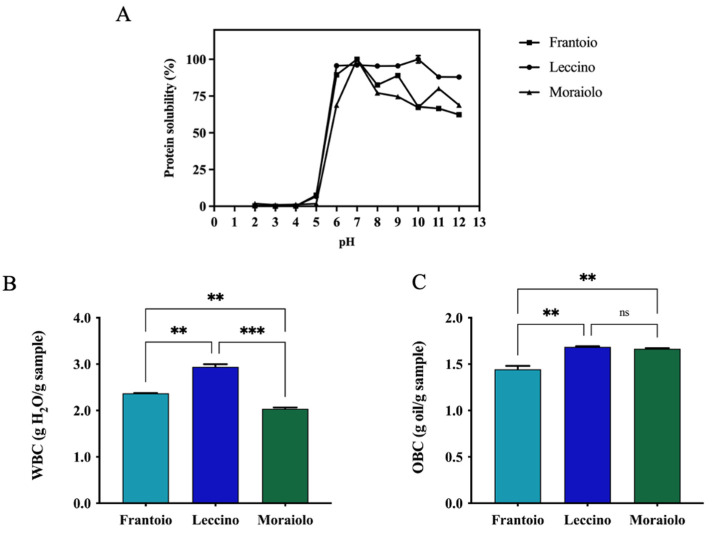
(**A**) Protein solubility. (**B**) Water-binding capacity (WBC). (**C**) Oil-binding capacity (OBC). Statistical analysis was performed by one-way ANOVA followed by Tukey’s pos-hoc test. (***) *p* < 0.001, (**) *p* < 0.01; ns, not significant. The data are represented as the means ± s.d. of three independent experiments.

Solubility is a functional property of food proteins that is commonly measured, and it is influenced by several factors including the pH, which is an essential environmental feature. For all samples, complete insolubility was observed across the pH 2 to 5 range. Protein solubility gradually increased when pH values were raised from 6 to In detail, *Frantoio*, *Leccino*, and *Moraiolo* proteins reached 89.4 ± 1.3%, 95.6 ± 0.4%, and 68.8 ± 0.7% solubility at pH 6 and 62.3% ± 0.4%, 87.9 ± 0.2%, and 68.8 ± 1.0% at pH 12, respectively. Particularly, the highest solubility was achieved at pH 7 for *Frantoio* and *Moraiolo* proteins and at pH 10 for *Leccino* ones. These results are in agreement with protein solubility of proteins isolated from other seeds, showing the best solubility in alkaline pH regions [42]. Another commonly valued property is the ability of extracted proteins to interact with water, which is dependent on their water-binding property. Many factors can influence WBC, including amino acid composition, protein conformation, surface polarity, and surface hydrophobicity [43]. In details, *Frantoio* and *Moraiolo* have a similar WBC equal to 2.3 and 2.0 g H_2_O/g sample; instead, *Leccino* has a higher WBC of 2.9 g H_2_O/g sample, which probably has a higher hydrophilic protein content (**, *p* < 0.01, Figure 3B). OBC corresponds to the amount of oil that a sample can absorb per unit of weight. Hence, *Frantoio* shows a reduced holding capacity compared with *Leccino* and *Moraiolo* (**, *p* < 0.01). In detail, *Leccino* and *Moraiolo* showed the same OBC values equal to 1.6 g oil/g sample and for *Frantoio* 1.4 g oil/g sample (Figure 3C).

### 3.4. Production of Olive Seed Protein Hydrolysates Using Alcalase and Papain

**Figure 4:**
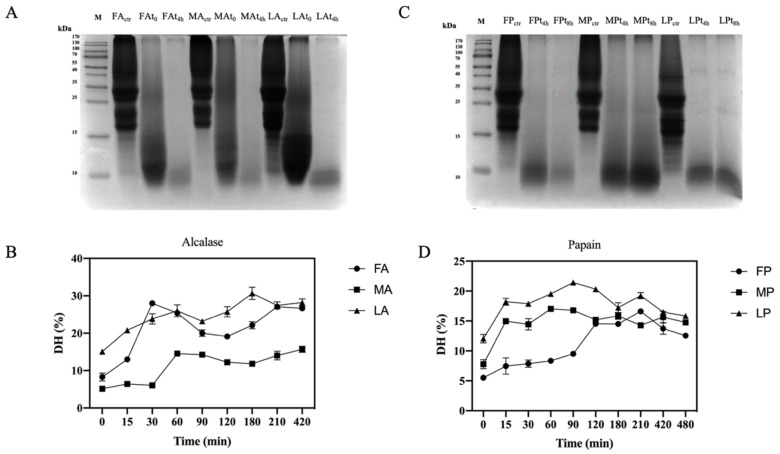
Digestion efficiency of olive seed total protein extract and degree of hydrolysis (DH) trend. (**A**) SDS-PAGE analysis of alcalase hydrolysates at different hydrolysis time points. FA, *Frantoio* digested by alcalase; MA, *Moraiolo* digested by alcalase; LA, *Leccino* digested by alcalase. (**B**) Alcalase DH at different time points. (**C**) SDS-PAGE analysis of papain hydrolysates at different hydrolysis time points. FP, *Frantoio* digested by papain; MP, *Moraiolo* digested by papain; LP, *Leccino* digested by papain. (**D**) Papain DH at different time points. The data are represented as the means ± s.d. of three independent experiments; each experiment was performed in triplicate.

The use of digestive enzymes is a technique commonly used for the digestion of proteins and the release of peptides by implementing their bio-accessibility. In this study, two food-grade enzymes, namely alcalase and papain, which have different cleavage properties, were used for enzymatic hydrolysis. Alcalase cleaves peptide bonds and involves the Phe, Tyr, Trp, and Lys carboxyl groups, while papain cleaves the peptide bonds of hydrophobic regions, including the amino acids Ala, Val, Leu, Ile, Phe, Trp, and Tyr [44]. The efficiency of the hydrolysis was evaluated comparing the OTPE profiles and the olive seed proteins digested by alcalase and olive seed proteins digested by papain. From the SDS-PAGE (Figure 4A,C), it appears evident that all proteins were completely hydrolyzed during the enzymatic process with both enzymes. For alcalase, we compared for each cultivar an untreated sample (before hydrolysis), a sample at time zero (immediately after the addition of the enzyme), and a sample after 4 h of hydrolysis. In the case of papain, we compared for each cultivar an untreated sample, a sample at 4 h, and a sample at 8 h of hydrolysis, respectively. The DHs using papain was lower than those obtained using alcalase (Table 3). Given the different cleavage properties, both the degree of hydrolysis and the peptides sequences generated are different. However, with the use of papain, the DH is the same for all cultivars, while *Leccino* hydrolyzed with alcalase has a lower DH than *Frantoio* and *Moraiolo*. In fact, we observed stronger signals after 4 h in the SDS-PAGE at the level of 10–15 kDa. As far as papain hydrolysis is concerned, a more intense signal at the level of 10–15 kDa compared to the other samples was observed in the *Moraiolo* sample. Both alcalase [43,44,45] and papain [43,45] DH results are in agreement with the result obtained on different matrices.

### 3.5. Peptidomics Characterization of Olive Seed Hydrolysates: Analysis of Medium- and Short-Sized Peptides

**Figure 5:**
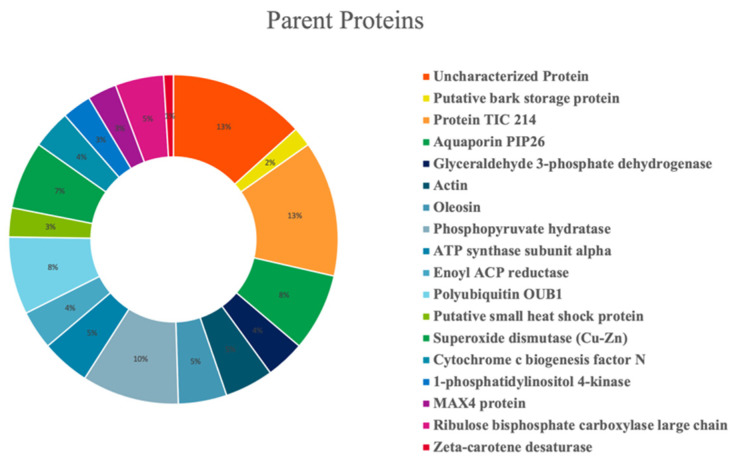
Percent distribution of identified peptides according to their parent proteins.

Olive seed hydrolysates were extensively investigated for the comprehensive characterization of both medium- and short-sized peptides. However, given the intrinsic differences between these two classes of peptides (mainly related to the length of the chain), two different strategies were employed. Medium-sized peptides were investigated using the conventional nanoHPLC-HRMS approaches borrowed from bottom-up proteomics. Short peptides, on the other hand, are incompatible with such analytical platform since loss could occur during sample preconcentration, and thus, they cannot be properly identified with the database approaches. Therefore, a dedicated metabolomics-based approach was employed [46]. After peptide validation, 104 and 491 medium- and short-sized peptide were annotated in the olive seed hydrolysates, respectively. Figure 5 shows the percent distribution of the peptides deriving from specific parent proteins. The olive seed hydrolysate contained peptides derived from several proteins, with a small prevalence of protein TIC 214, a protein involved in protein precursor import into chloroplasts (13%) and a phosphopyruvate hydratase (10%). Other parent proteins include aquaporin PIP26 (8%), polyubiquitin OUB1 (8%), and superoxide dismutase (Cu-Zn) (7%)

To broaden the investigation to potential bioactivities, the short peptides were ranked by the tool PeptideRanker (http://bioware.ucd.ie/~compass/biowareweb/Server_pages/peptideranker.php; accessed on 4 May 2022). At the end of this process, only those peptides showing score values higher than 0.6 were considered as potentially bioactive and submitted to BIOPEP search (http://www.uwm.edu.pl/biochemia/index.php/pl/biopep/; accessed on 4 May 2022), an open-access database that allows to hypothesize potential biological activities of peptides based on the presence of some short amino acid sequences (Table 4). The presumed biological activities included the inhibition of DPP-IV and the antioxidant property. Of all the short peptides identified, 77 with potential biological activity were identified. Most of these bioactivities are predominantly provided by short sequences of two or three amino acids included in their structures.

### 3.6. Antioxidant Activity of Olive Seed Hydrolysates

#### 3.6.1. Direct Radical Scavenging Activity of Olive Seed Hydrolysates by DPPH Assay

**Figure 6:**
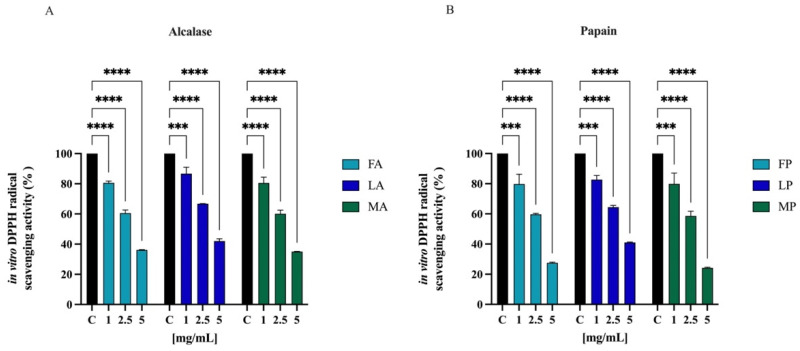
Direct radical scavenging activity of olive seed hydrolysates rom *Frantoio* (F), *Leccino* (L), and *Moraiolo* (M) obtained using alcalase (A) (**A**) and papain (P) (**B**) by DPPH assay. Data represent the mean ± s.d. of six independent experiments, and each experiment was performed in triplicate. All the data sets have been analyzed by two-way ANOVA. (****) *p* < 0.0001, (***) *p* < 0.001.

The radical scavenging activities of DPPH have regularly been used to evaluate the antioxidant potentials of natural compounds to act as free radical scavengers [47]. Figure 6 shows the DPPH radical scavenging activity of olive seed hydrolysates in the range of concentrations 1.0–5.0 mg/mL. FA, LA, and MA scavenged the DPPH radical by 19.5 ± 1.1%, 13.4 ± 4.3%, and 19.5 ± 3.8% at 1.0 mg/mL, respectively; they scavenged the DPPH radical by 39.5 ± 2.1%, 33.2 ± 0.1%, and 39.8 ± 2.4% at 2.5 mg/mL, respectively. For 5 mg/mL, the DPPH radical is reduced by 63.9 ± 0.2%, 58.0 ± 1.4%, and 65.0 ± 0.1% for FA, LA, and MA, respectively. FP, LP, and MP scavenged the DPPH radical by 20.1 ± 6.5%, 17.4 ± 2.7%, and 20.2 ± 7.1% at 1.0 mg/mL respectively, and they scavenged the DPPH radical by 40.4 ± 2.1%, 35.6 ± 1.2%, and 41.4 ± 3.1% at 2.5 mg/mL, respectively. For 5 mg/mL, the DPPH radical is reduced by 72.5 ± 0.5%, 59.0 ± 0.3%, and 75.7 ± 0.4% for FP, LP, and MP, respectively.

#### 3.6.2. Direct Radical Scavenging Activity of Olive Seed Hydrolysates by ABTS Assay

**Figure 7:**
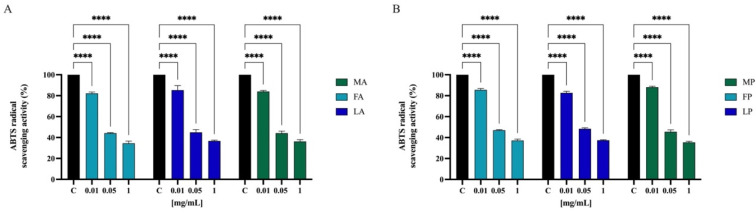
Direct radical scavenging activity of olive seed hydrolysates rom *Frantoio* (F), *Leccino* (L), and *Moraiolo* (M) obtained using alcalase (A) (**A**) and papain (P) (**B**) by ABTS assay. Data represent the mean ± s.d. of six independent experiments, and each experiment was performed in triplicate. All the data sets have been analyzed by two-way ANOVA. (****) *p* < 0.0001.

To further confirm the direct antioxidant property, the ABTS free scavenging activity of olive seed hydrolysates was determined at 0.01, 0.05, and 1.0 mg/mL. As indicated in Figure 7, FA, LA, and MA scavenged the ABTS radical by 17.8 ± 1.3%, 14.7 ± 4.3%, and 16.0 ± 0.9% at 0.01 mg/mL, respectively, and they scavenged the ABTS radical by 55.8 ± 0.5%, 55.1 ± 2.6%, and 55.8 ± 1.9% at 0.05 mg/mL, respectively. Finally, FA, LA, and MA scavenged the ABTS radical by 64.3 ± 0.8%, 64.6 ± 0.9%, and 65.3 ± 1.6% at 0.5 mg/mL, respectively. Hydrolysates with papain show a similar trend. FP, LP, and MP scavenged the ABTS radical by 14.5 ± 1.3%, 17.3 ± 1.4%, and 11.9 ± 0.9% at 0.01 mg/mL, respectively; they scavenged the same radical by 53.0 ± 0.5%, 51.7 ± 0.9%, and 54.5 ± 1.8% at 0.05 mg/mL, respectively. Lastly FP, LP, and MP scavenged the ABTS radical by 65.1 ± 1.2%, 64.8 ± 0.5%, and 65.3 ± 0.8% at 1.0 mg/mL, respectively.

#### 3.6.3. Ferric-Reducing Antioxidant Power (FRAP) Activity

**Figure 8:**
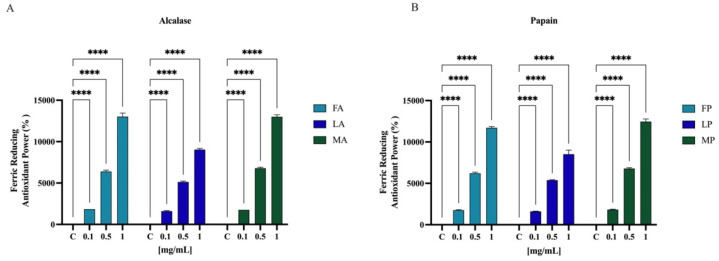
Antioxidant power evaluation by ferric reducing antioxidant power (FRAP) of olive seed hydrolysates from *Frantoio* (F), *Leccino* (L), and *Moraiolo* (M) obtained using Alcalase (A) (**A**) and Papain (P) (**B**). Data represent the mean ± s.d. of six determinations performed in triplicate. All the data sets have been analyzed by Two-way ANOVA. (****) *p* < 0.0001.

Results showed that FA, LA, and MA increased the FRAP by radical by 1850.0 ± 1.8%, 1612.5 ± 62.9%, and 1750.5 ± 2.0% at 0.1 mg/mL, respectively. For 0.5 mg/mL, the FRAP levels are increased by 6387.5 ± 160.0%, 5125.0 ± 104.0%, and 6787.5 ± 118.1% for FA, LA, and MA, respectively. Finally, FA, LA, and MA tested at 1.0 mg/mL improved the FRAP by 13,025.0 ± 429.1%, 9037.5 ± 143.6%, and 13,025.0 ± 241.5%, respectively (Figure 8A). Papain hydrolysates also showed a significant increased the FRAP levels. In Figure 8B, results show that FP, LP, and MP tested at 0.1 mg/mL improved the FRAP by 1750.0 ± 70.7%, 1612.5 ± 25.0%, and 1825.0 ± 50.0%, respectively. For 0.5 mg/mL, the FRAP levels are increased by 6212.5 ± 131.4%, 5375.0 ± 64.5%, and 6787.5 ± 118.1% for FP, LP, and MP, respectively. Finally, FP, LP, and MP tested at 1.0 mg/mL improved the FRAP by 11,712.5 ± 149.3%, 8512.5 ± 495.6%, and 12,462.5 ± 311.9%, respectively.

#### 3.6.4. PH and AH Hydrolysates Decrease the H_2_O_2_-Induced ROS and Lipid Peroxidation Levels in Intestinal Caco-2 Cells

**Figure 9:**
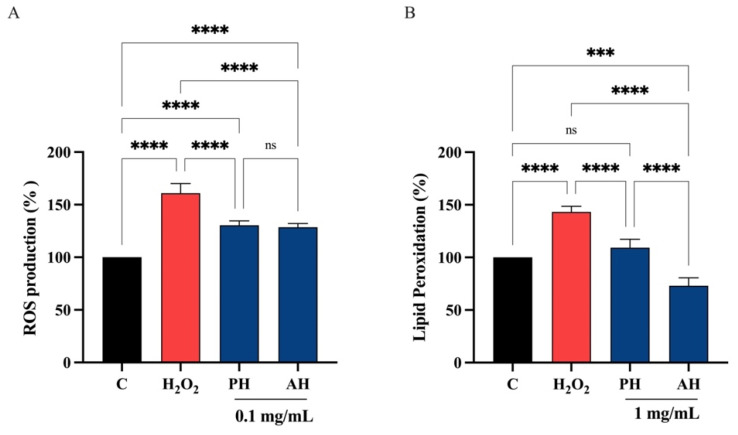
(**A**) Modulation of intracellular H_2_O_2_-induced ROS levels after the pretreatment with PH and AH hydrolysates. Modulation of H_2_O_2_-induced lipid peroxidation after pretreatment with PH and AH hydrolysates (**B**). Data represent the mean ± s.d. of six independent experiments, and each experiment was performed in triplicate. All data sets were analyzed by one-way ANOVA followed by Tukey’s post hoc test. C, control sample; ns, not significant; (***) *p* < 0.001, (****) *p* < 0.0001.

Considering the comparable ability of each peptide hydrolysate from the three different cultivars obtained with the two enzymes, PH and AH samples were prepared mixing peptides from *Frantoio*, *Leccino*, and *Moraiolo* obtained using papain (PH) and alcalase (AH), respectively. PH and AH were used for evaluating their antioxidant activity on human intestinal Caco-2 cells. To achieve this objective, the fluorometric intracellular ROS assay was performed. Preliminary MTT experiments were carried out for investigating the concentrations of the olive seed hydrolysates obtained using alcalase or papain that may possibly impair the Caco-2 cell viability. After a 48 h treatment, no significant cell mortality was detected up to 5 mg/mL versus untreated cells (C) (Figure S1). Figure 9 clearly shows that the H_2_O_2_ treatment leads to an increase of intracellular ROS levels by 161.0 ± 9.10% versus the control cells, which were mitigated by pre-treatment with PH and AH hydrolysates that restore the ROS up 130.4 ± 4.24% and 128.5 ± 3.60%, respectively, at 0.1 mg/mL (Figure 9A). In addition, the capacity of PH and AH hydrolysates to modulate the H_2_O_2_-induced lipid peroxidation in human intestinal Caco-2 cells was assessed by the MDA measurement. In accordance with the observed increase ROS after the H_2_O_2_ treatment, a noticeable increase of the lipid peroxidation was observed up to 143.2 ± 5.44% versus the control cells. Moreover, our results showed that the pretreatment with PH and AH hydrolysates (1 mg/mL) resulted in a reduction in MDA levels up to 109.2 ± 7.95% and 73.0 ± 7.64%, respectively, restoring the lipid peroxidation baseline levels (Figure 9B).

### 3.7. Antidiabetic Activity of Olive Seed Hydrolysates

#### 3.7.1. In Vitro and Cellular DPP-IV Inhibitory Activity of Olive Seed Hydrolysates

**Figure 10:**
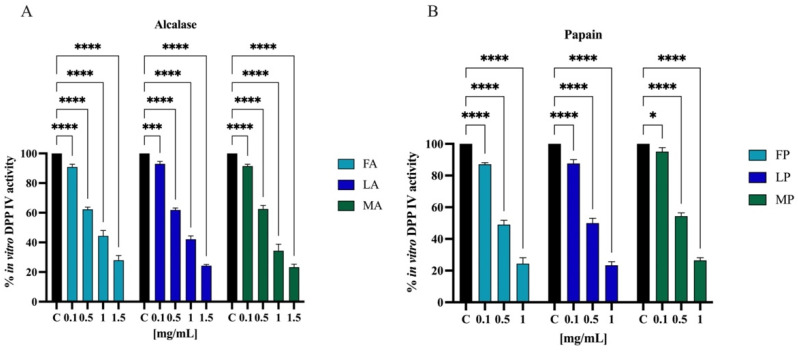
Effect of olive seed hydrolysates obtained from *Frantoio* (F), *Leccino* (L), and *Moraiolo* (M) using alcalase (A) (**A**) and papain (P) (**B**) on the in vitro DPP-IV activity, respectively. The data points represent the averages ± s.d. of four independent experiments, and each experiment was performed in triplicate. All data sets were analyzed by two-way ANOVA followed by Tukey’s post hoc test. Ns, not significant; C, control sample (H*_2_*O). (*) *p* < 0.5, (***) *p* < 0.001, (****) *p* < 0.0001.

Biochemical experiments were realized to assess the inhibitory activity of olive seed hydrolysates against human recombinant DPP-IV enzyme. The enzymatic reaction was monitored by measuring the fluorescence signals at 465 nm after excitation at 350 nm due to the release of the free-AMC group after the cleavage of the fluorescent substrate H-Gly-Pro-AMC by DPP-IV. The activity of separate olive seed hydrolysates obtained from the three cultivars using alcalase was screened at the final concentrations of 0.1, 0.5, 1.0, and 1.5 mg/mL. Figure 10A suggests that the FA sample reduced the DPP-IV activity by 9.8 ± 1.8%, 37.8 ± 1.5%, 55.7 ± 3.7%, and 72.1 ± 3.1%. The LA sample lowered the DPP-IV activity by 7.1 ± 1.6%, 38.2 ± 1.3%, 57.9 ± 2.2%, and 75.8 ± 0.8%. Finally, the MA sample decreased the DPP-IV activity by 8.6 ± 1.3%, 37.5 ± 2.4%, 65.7 ± 4.3%, and 76.7 ± 1.9%. Similarly, the activity of separate olive seed hydrolysates obtained from the three cultivars using papain was screened at the final concentrations of 0.1, 0.5, and 1.0 mg/mL. Figure 10B indicates that the FP sample reduced the DPP-IV activity by 12.9 ± 1.0%, 51.0 ± 2.6%, and 75.7 ± 3.7%. The LP sample reduced the DPP-IV activity by 12.4 ± 2.3%, 50.1 ± 2.3%, and 76.6 ± 2.2%. Lastly, the MP sample inhibited the DPP-IV activity by 4.9 ± 2.4%, 45.7 ± 2.1%, and 73.6 ± 1.6%, respectively.

**Figure 11:**
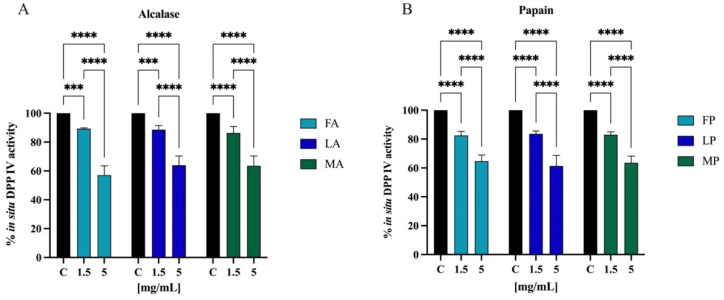
Effect of olive seed hydrolysates obtained from *Frantoio* (F), *Leccino* (L), and *Moraiolo* (M) using alcalase (A) (**A**) and papain (P) (**B**) on DPP-IV activity expressed by Caco-2 cells. The data points represent the averages ± s.d. of four independent experiments, and each experiment was performed in triplicate. All data sets were analyzed by two-way ANOVA followed by Tukey’s post hoc test. (***) *p* < 0.001, (****) *p* < 0.0001.

The most frequently used model of human intestinal enterocytes is the human intestinal Caco-2 cell line. Numerous intestinal enzymes engaged in food digestion are expressed on the surface of Caco-2 cells, including DPP-IV Since both alcalase- and papain-derived hydrolysates have shown to express DPP-IV inhibitory activity in vitro, the validated Caco-2 cell-based DPP-IV activity assay was used to test the effects of both each sample on DPP-IV activity at cellular levels. Their activity was screened at the final concentration of 1.5 and 5.0 mg/mL. Figure 11A shows that FA inhibited the cellular DPP-IV activity by 10.7 ± 0.6% and 42.9 ± 6.5%, LA by 11.4 ± 2.8% and 36.2 ± 6.4%, and MA by 13.7 ± 4.5% and 36.4 ± 6.8% at 1.5 and 5.0 mg/mL, respectively. Regarding hydrolysates obtained using papain, FP inhibited cellular DPP-IV activity by 17.5 ± 2.7% and 35.3 ± 4.2%, LP by 16.4 ± 2.0% and 38.7 ± 7.2%, and MA by 17.0 ± 2.0% and 36.5 ± 4.6% at 1.5 and 5.0 mg/mL, respectively (Figure 11B).

**Figure 12:**
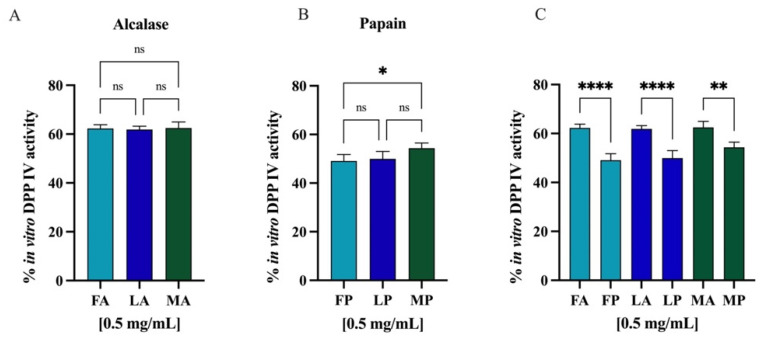
(**A**) Comparison between the *cultivars* hydrolyzed by alcalase, (**B**) the *cultivars* hydrolyzed by papain, and (**C**) the same cultivars hydrolyzed with different enzymes. Ns, not significant; (*) *p* < 0.5, (**) *p* < 0.01, (****) *p* < 0.0001.

Comparing the DPP-IV inhibitory activity of the hydrolysates obtained from different *cultivars* but hydrolyzed with the same enzyme, no statistically significant differences were observed (Figure 12A,B). However, regarding the same *cultivar* but hydrolyzed with different enzymes, alcalase and papain, comparison revealed significative differences (Figure 12C), and in all the cases, the papain hydrolysates showed stronger inhibitory activity than alcalase samples (*p* < 0.0001 for *Frantoio* and *Leccino* and *p* < 0.01 for *Moraiolo*). Thus, PH and AH prepared mixing the peptides from *Frantoio*, *Leccino*, and *Moraiolo* obtained using papain and alcalase, respectively, were used for deeper evaluation of their in vitro anti-diabetic effects.

#### 3.7.2. STC-1/Caco-2 Co-Culture System and Assessment of Olive Seed Hydrolysates Capacity to Modulate Stability and Secretion of GLP-1 Hormone

**Figure 13:**
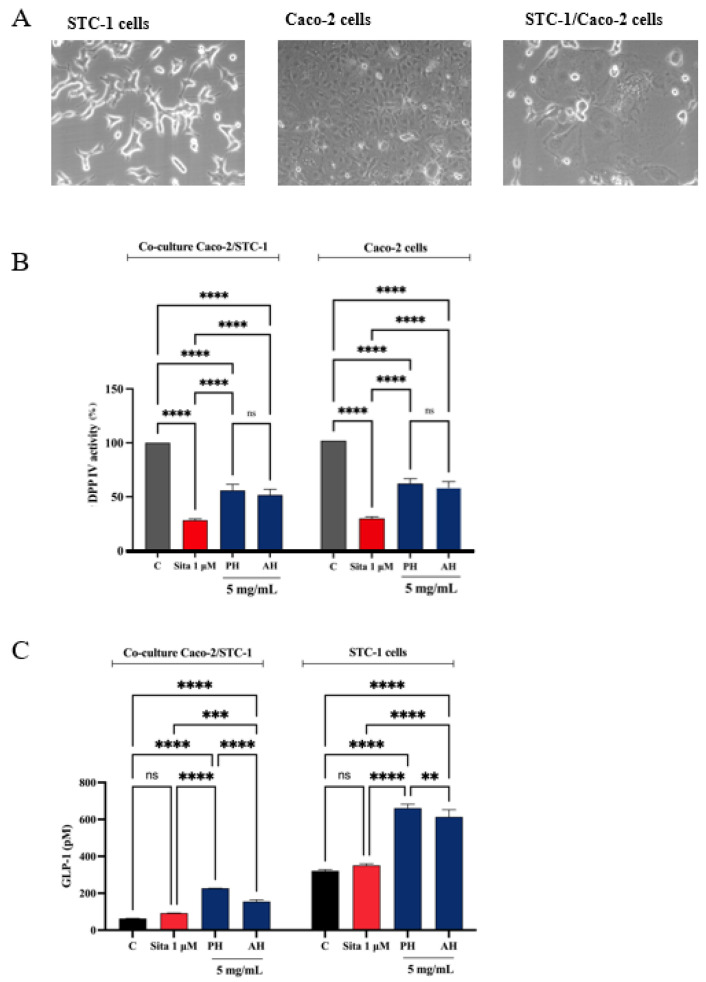
(**A**) Microscope cell images obtained by AxioCam MRc 5 Zeiss after 48 h from seeding. (**B**) Effect of PH and AH on the in situ DPP-IV activity in both co-culture Caco-2/STC-1 and Caco-2 alone cells, respectively. (**C**) Effect of PH and AH on the GLP-1 concentration levels (expressed as pM) in both co-culture Caco-2/STC-1 and STC1-alone cells, respectively. Data points represent averages ± s.d. of three independent experiments in duplicate. All data sets were analyzed by two-way ANOVA followed by Tukey’s post hoc test. C, control sample; Sita, sitagliptin; ns, not significant. (**) *p* < 0.01, (***) *p* < 0.001, and (****) *p* < 0.0001 vs. control (C).

A co-culture system was developed using Caco-2 enterocytes cells and STC-1 enteroendocrine cells to evaluate whether the treatment with potential DPP-IV inhibitors increased the stability of the GLP-1 hormone and whether they increased GLP-1 secretion (Figure 13A). Given the complexity of the cellular system and considering their similar ability in inhabiting the DPP-IV enzymes in both cell-free and cell-based conditions, we decided to directly test PH and AH samples, respectively. Therefore, both the co-culture and the STC-1 cells were treated with 5 mg/mL of each mixture (the concentration that determined the highest DPP-IV inhibition on Caco-2 cells, as highlighted in Figure 11) or sitagliptin 1 µM for 1 h, and the supernatant was collected to proceed with the GLP-1 detection. Before proceeding with the treatments, the cell viability of the co-culture was verified. After a 48 h treatment, no significant impairment of cellular viability was detected versus untreated cells (C) (Figure S2). Figure 13B indicates that PH inhibited the cellular DPP-IV activity by 44.1 ± 5.6% and AH by 48.3 ± 5.2% in the co-culture system, respectively, and 71.6 ± 1.2% for sitagliptin. Moreover, PH inhibited the cellular DPP-IV activity by 38.6 ± 4.3% and AH by 42.4 ± 5.4% in Caco-2 cells alone and 70.3 ± 1.4% for sitagliptin. The treatment with sitagliptin (1 µM) and PH and AH hydrolysates led to an increase of GLP-1 levels versus the control cells (Figure 13C). In detail, after 1 h treatment with PH and AH hydrolysates, the GLP-1 levels in the co-culture system reached 154.9 ± 9.6 pM and 226.7 ± 0.3 pM, respectively, at 5 mg/mL and 92.26 ± 1.1 pM for sitagliptin (Figure 13C). Statistical analysis clearly revealed that any significant differences were observed in the GLP-1 level modulation between sitagliptin and untreated cells, indicating that the reference compound through the inhibition of DPP-IV improves the GLP-1 stability, but it is unable to modulate its production in the co-culture system. On the contrary, both PH and AH improved the GLP-1 secretion in both cellular systems, suggesting a hypoglycemic activity occurring with a mechanism of action that is different than that of sitagliptin. Indeed, together with inhibition of DPP-IV, PH is more effective than AH in increasing the GLP-1 stability and secretion in the co-culture STC1/Caco-2 system (Figure 13C). This result is also confirmed when STC-1 alone was treated with both PH and AH (Figure 13C). In fact, results clearly show that, similarly, both olive seed hydrolysates increase the GLP-1 secretion by STC-1 cells. In detail, PH and AH hydrolysates lead to an increase of GLP-1 levels up to 660.7 ± 21.9 pM and 613.4 ± 39.1 pM, respectively (Figure 13C).

## 4. Discussion

Nowadays, food bioactive peptides represent a hot and challenging topic of research, and a great deal of evidence clearly underlines that food side-streams and/or by-products may be considered valuable sources of bioactive peptides with health-promoting properties. In this particular context, this study provides new evidences that olive seed proteins could be recovered and successfully exploited for the production of food bioactive peptides. To take into account the compositional variability, each seed sample was designed and built as a mix of several batches of olive fruits. With this approach, it was possible to study the protein composition of an average sample for each of the three cultivars. Furthermore, according to the time of collection, the sample was also suitable to reflect the composition of the seeds of the olive fruits used for the milling process to produce virgin olive oils. To our knowledge, such a study has never been performed on the seeds of the olive fruit, particularly on the seeds of these typical Tuscan cultivars. Indeed, starting from three different cultivars of olive seed, *Frantoio*, *Moraiolo*, and *Leccino*, proteins have been isolated using optimized conditions and characterized from physico-chemical and functional point of views. Hence, protein hydrolysates have been produced by enzymatic hydrolysis, which has become a widely used biotechnological process to obtain food protein hydrolysates endowed with biological activities [48]. Alcalase and papain, two commercially and well-known food-grade enzymes, have been employed to hydrolysate these proteins. Comparing the DH %, alcalase was about 2-fold more efficient than papain in hydrolyzing the olive seed proteins extracted from *Frantoio* and *Leccino*. The more efficient ability of alcalase than papain to hydrolyze plant-derived proteins is in complete agreement with previous study [49]. On the contrary, both enzymes displayed the same ability to hydrolyze the *Moraiolo* proteins, indicating that alcalase was less efficient in digesting these proteins than those extracted from *Frantoio* and *Leccino*.

Since the biological activity of a specific protein hydrolysate is strictly associated to its chemical composition, the identification of olive seed peptide sequences was assessed by applying the most updated analytical techniques. Hence, the characterization of the medium-sized peptide revealed the presence of 104 peptides within the hydrolysates belonging to several parent proteins, i.e., protein TIC 214, a protein involved in protein precursor import into chloroplasts (13%), phosphopyruvate hydratase (10%), aquaporin PIP26 (8%), polyubiquitin OUB1 (8%), and superoxide dismutase (Cu-Zn) (7%).

Unlike medium-sized, the short-sized peptides identification is a challenging topic for several reasons [50]. Standard proteomics database search engines cannot efficiently annotate short-sized peptides since their short sequence cannot be confidently associated with single proteins [51]. Moreover, the short sequences generate mostly singly charged ions, which are non-compatible with proteomics workflows and generate noisier MS/MS spectra. Finally, the fragmentation pathways of short peptide sequences are more strongly affected by the nature of the single amino acid that constitutes the sequences [46].

Nevertheless, applying a high-resolution mass spectrometry-based suspect screening approach, these particular issues were overcome and used to identify, for the first time, the sequence of short peptides within olive seed protein hydrolysates (Table S1). Notably, the olive seed hydrolysates contain 491 different short-sized peptides containing 2–4 amino acid residues. The parallel analysis of medium and short peptide sequences using the BIOPEP database allowed us to identify many sequences containing some known antioxidant and anti-diabetic characteristics (Table 4), respectively.

### 4.1. Olive Seed Hydrolysates Exert Direct and Cellular Antioxidant Activity

To evaluate the radical scavenging activity of olive seed hydrolysates, DPPH, ABTS, and FRAP assays were employed since these tests are widely applied to screen and evaluate the ability of natural compounds to act as free radical scavengers or hydrogen donors. Results clearly indicate that all the olive seed hydrolysates produced using both alcalase and papain exert a direct antioxidant activity being able to scavenge DPPH radicals in the range 1–5 mg/mL (Figure 6) and ABTS radicals in the range 0.01–1 mg/mL (Figure 7). Thus, olive seed hydrolysates are more active as ABTS than DPPH radical scavengers. This result may be explained considering that ABTS assay is based on the generation of a blue/green ABTS·+, which is applicable to both hydrophilic and lipophilic antioxidant systems, whereas DPPH assay uses a radical dissolved in organic media and is, therefore, applicable to hydrophobic systems [52]. Hence, using ABTS assay, the antioxidant contribution of total hydrophobic and hydrophilic peptides was measured, whereas using DPPH test, the antioxidant contribution of only hydrophobic peptides was detected. In addition, the same peptide mixtures improve the ability to reduce the Fe(III) at Fe(II) in the range 0.1–1 mg/mL (Figure 8).

Many physical-chemical factors may influence the ability of peptides to exert antioxidant activity. In fact, although certain aspects of the structure–function relationship of antioxidant peptides are still poorly understood [53], it has been suggested that chain length, amino acid type, amino acid composition, and amino acid sequence, which is the location of specific amino acids in a peptide chain, may be critical issues for exerting the antioxidant property [54,55,56]. In this peculiar context, all the tested olive seed hydrolysates are enriched of short peptides, which are often considered potent antioxidants, and they contain hydrophobic amino acids (such as Leu or Val) in their N-terminal regions, nucleophilic sulfur-containing amino acid residues (Cys and Met), or aromatic amino acid residues (Phe, Trp, and Tyr) and/or the imidazole ring-containing His, which are generally found to possess strong antioxidant properties [54]. The direct antioxidant ability of olive seed hydrolysates is in agreement with previous investigation in which it has been shown that olive seed hydrolysate obtained using alcalase displayed the highest antioxidant capacity compared to the hydrolysates obtained using neutrase, thermolysin, flaovourzyme, and PTN [57]. In addition, olive seed hydrolysates show a similar ability to soybean (obtained using pepsin and trypsin) and hempseed (produced using pepsin) hydrolysates, respectively, to scavenge DPPH radicals [54,55], whereas they are more active than that of a hempseed protein hydrolysate obtained by co-digesting the proteins with pepsin and pancreatin, which has shown to be a poor scavenger of DPPH, i.e., about 4% [58]. Instead, rice bran protein hydrolysates obtained after the hydrolysis of the proteins with alcalase displayed a DPPH radical scavenging activity of about 32% at 20 mg/mL [59]. Finally, fish and chicken bone hydrolysates obtained using trypsin showed an antioxidant activity of approximately 15 and 10%, respectively, at 5.0 mg/mL [60].

To validate and confirm the antioxidant activity of olive seed hydrolysates, their effects were assessed at cellular level using human intestinal Caco-2 cells. Considering that *Frantoio*, *Leccino*, and *Moraiolo* protein-derived hydrolysates obtained using alcalase show the same direct antioxidant activity, a mixture of the three hydrolysates (1:1:1) was prepared and named as alcalase hydrolysate (AH) (Figure 6A, Figure 7A and Figure 8A). Similarly, a mixture of the three hydrolysate powders obtained using papain (1:1:1) was prepared and named as papain hydrolysate (PH) (Figure 6B, Figure 7B and Figure 8B). Indeed, our findings underline a dramatic increase of intracellular ROS when Caco-2 cells are treated with H_2_O_2_ (*p* < 0.0001), but the pre-treatment with AH and PH significantly and equally protected the human intestinal cells, thus restoring the ROS level to basal conditions and confirming their good ability to act as natural antioxidants (Figure 9A). High oxidative stress results in significant damage to human cells by altering proteins, lipids, and DNA, leading to several simultaneous processes that may culminate in pathological conditions involved in the progression of cardiovascular disease. Lipid of cellular membranes are susceptible to oxidative attack, typically by ROS, resulting in a well-defined chain reaction with the production of end products such as malondialdehyde (MDA) and related compounds known as TBA reactive substances (TBARS). Notably, in agreement with the observed increase of ROS after the H_2_O_2_ treatment, a significant increase of the lipid peroxidation at cellular level was observed (*p* < 0.0001). In addition, the pre-treatment of Caco-2 cells with both AH and PH hydrolysates determine a significant reduction of lipid peroxidation, and statistical analysis revealed that AH is more efficient than PH (*p* < 0.0001), being able to reduce the lipid peroxidation even under basal condition (*p* < 0.001). These results are totally in line with the effect of soybean and hempseed hydrolysates in the modulation of intracellular ROS and lipid peroxidation levels after the H_2_O_2_ stimulation of human intestinal Caco-2 and human hepatic HepG2 cells, respectively [54,55].

### 4.2. Olive Seed Hydrolysates Inhibit DPP-IV Activity and Improve the Stability and Secretion of GLP-1

Protein hydrolysate, which is characterized by a high heterogeneous composition, may possess more than one biological activity, therefore exerting a multifunctional behavior. Indeed, beside their antioxidant property, olive seed hydrolysates inhibit the DPP-IV activity in a cell-free system in which human recombinant enzyme was applied (Figure 10) and in a cell-based assay in which human intestinal Caco-2 cells were employed (Figure 11). Flaxseed, rapeseed, sunflower, sesame, soybean, hempseed, whey, and casein hydrolysates generated using alcalase showed the capacity to reduce the DPP-IV activity using a cell-free in vitro system [61]. Specifically, whey hydrolysates showed inhibition by 42%, followed by the rapeseed hydrolysates that reached a value of 30%, while the other hydrolysates inhibited the DPP-IV activity by around 20% at a concentration of 10 mg/mL. In addition, chickpea and collagen hydrolysates obtained using papain reached in vitro DPP-IV inhibition by 45.5% at 0.1 mg/mL and by 55.2% at 1 mg/mL, respectively [61,62]. In agreement with evidence from the literature, this study confirms that papain-derived hydrolysates is more active than alcalase-derived peptide mixture in decreasing the DPP-IV activity (Figure 12).

Our results clearly indicate that olive seed hydrolysates are about 10-fold more active in a cell-free conditions than in cell-based system. This difference may be explained considering that when the peptide mixtures encounter human intestinal Caco-2 cells, they may be further hydrolysated by active peptidase, which are expressed on their cellular membranes [63], and/or they are concomitantly up-taken at intracellular levels. Overall, this behavior is in agreement with previous studies on hydrolysates from *Arthrospira platensis* (spirulina) and *Chlorella pyrenoidosa* proteins [63,64]. In particular, spirulina hydrolysate obtained with pepsin inhibited DPP-IV activity by 64% in a cell-free system and 34% in Caco-2 cells, while the inhibition of hydrolysates obtained with trypsin reached 72% and 41% in a cell-free system and Caco-2 cells at a dose of 5.0 mg/mL, respectively [64,65]. Other studies have also shown the capacity of hempseed and soybean protein hydrolysates to inhibit DPP-IV activity. In particular, 1.0 mg/mL of peptic hempseed hydrolysates inhibited the DPP-IV activity by 32% in vitro and 22% in Caco-2 cells [37], while DPP-IV activity was inhibited by 2.5 mg/mL of peptic soybean hydrolysates by 31% and 11% in vitro and in Caco-2 cells, respectively.

The possibility to establish a reasonable structure–function relationship of the DPP-IV inhibitory property of olive seed peptide mixtures is impaired by the fact that the bioactivity of a protein hydrolysate depends strictly on its total composition, including inactive and active species and possible synergistic or antagonist effects. In this peculiar context, olive seed hydrolysates are rich in hydrophobic short-chain peptides sequences containing a Pro residue within their sequences, which is located at the first, second, third, or fourth N-terminal position. Moreover, the Pro residue is flanked by Leu, Val, Phe, Ala, and Gly (Table 4 and Table S1).

It is doubtless that in the field of food bioactive peptides, DPP-IV inhibitory activity is among the most studied health-promoting effects. Notwithstanding, the evaluation of GLP-1 stability as a consequence of DPP-IV inhibition is completely underestimated. In order to fill this gap, in this study, a co-culture system employing Caco-2 enterocytes cells and intestinal STC-1 endocrine cells was developed (Figure 13A). Notably, intestinal Caco-2 cells express DPP-IV enzymes on their surfaces [6], whereas STC-1 cells produce and secrete GLP-1 hormone, which is the physiological DPP-IV substrate [66]. Thus, combining both cellular systems, it was possible to dynamically and directly assess the effect of DPP-IV inhibition exerted by olive seed hydrolysates on the GLP-1 stability and production. The experiments were performed using sitagliptin as reference compound. Moreover, in comparing the activity of the same cultivar but hydrolyzed with the two different enzymes (alcalase and papain), significative differences were observed (Figure 12), and in all the cases, the papain hydrolysates showed stronger inhibitory activity compared with the alcalase derived peptide mixtures (*p* < 0.0001 for *Frantoio* and *Leccino* and *p* < 0.01 for *Moraiolo*); the total PH and AH samples were investigated in the co-culture systems. In more detail, findings confirmed that similarly to sitagliptin, both PH and AH reduced the DPP-IV activity in both Caco-2 cells alone and Caco-2/STC-1 cells, respectively (Figure 13B). Hence, in agreement with DPP-IV inhibition in the co-culture Caco-2/STC-1 cells, sitagliptin slightly increases the GLP-1 levels even though this augmentation is not statically significative (Figure 13C). Interestingly, both AH and PH greatly increase the production of GLP-1 in the same co-culture systems, and PH is more effective than AH peptides (*p* < 0.0001, Figure 13C). These results clearly suggest an additional and different mechanism of action of olive seed peptides of sitagliptin through which they may exert the potential anti-diabetic effects. In order to better address this peculiar issue, sitagliptin and both AH and PH were tested on STC-1 alone. As shown in Figure 13C, it is clear that sitagliptin is ineffective on GLP-1 levels, whereas both AH and PH greatly improve the GLP-1 production and secretion, and also in these cells, PH is confirmed more active than AH in the modulation of GLP-1 levels (*p* < 0.1). Taking together these results, it is certainly important to underline that similarly to olive seed hydrolysates, other food bioactive peptides may display anti-diabetic activity with a complementary mechanism of action targeting, therefore not only impacting DPP-IV activity but directly modulating the GLP-1 production.

## 5. Conclusions

Combining biochemical and cellular techniques, our findings demonstrated that the olive seed hydrolysates obtained digesting olive seed proteins with both alcalase (AH) and papain (PH) show multifunctional activities, as they are able to exert both antioxidant and DPP-IV inhibitory activity and to increase the GLP-1 production. In addition, combining both intestinal Caco-2 and STC-1 cells through the development of the co-culture systems, we have clearly demonstrated how the dynamic DPP-IV inhibition (expressed by Caco-2 cells) by both AH and PH peptides positively reflects on the stability of GLP-1 expressed by STC-1 cells. Surprisingly, we have also demonstrated that both AH and PH peptides enhance the GLP-1 production by STC-1 cells with a mechanism of action that is different from that of sitagliptin.

Indeed, this study provides new evidences that besides their exploitation as energy source (biomass) production, olive seed peptides obtained from residual materials of table olive and olive oil production could be recovered and exploited as valuable ingredients for upgrade application, i.e., functional foods and/or dietary supplement developments. In addition, for a large-scale production of these hydrolysates, the hydrolysis process might be performed using immobilized enzymes. This technique that is used in the food, chemical, pharmaceutical, cosmetic, and medical device industries provides many advantages in term of costs, reproducibility, and also sustainability. Another important issue that need to be elucidated further regard the potential use of pectinolytic enzyme mixtures as a pretreatment approach for improving the extraction yields of proteins from olive seeds. In conclusion, this study can be considered the first step through which in vitro and cellular screening allow to assess and molecular characterize the mechanism through which olive seed peptides may exert antioxidant and anti-diabetic activity; certainly, other in vivo studies on suitable animal models are needed to obtain the “*proof of concept*” regarding the health promoting activity of both hydrolysates. Notably, upon the administration of hydrolysates on an animal model, the ability of peptides to exert antioxidant and anti-diabetic effects targeting DPP-IV and GLP-1 will be evaluated. These results can be useful for the development of nutraceutical and functional foods for preventing diabetic condition and oxidative stress.

## Figures and Tables

**Table 1:** Alcalase and papain enzymes hydrolysis conditions: temperature (°C), hydrolysis time, E:S ratio, and pH.

Enzyme	Temperature (°C)	Hydrolysis Time	Enzyme: Substrate Ratio	pH
Alcalase	50	4 h	0.15 UA/g	8.5
Papain	65	8 h	100 UA/g	7

**Table 2:** Description of buffers used for protein extraction and yield in mg/mL.

ID	Extraction Buffer	(mg/mL)
U	UREA 8 M, 1% CHAPS, 0.1% DTT, NH_4_HCO_3_	9.2
T	0.1 M Tris (pH 7.5), 0.5 M NaCl, 0.5% SDS, 0.1% DTT	1.9
U/T	UREA 6 M, 0.1 M Tris-HCl (pH 8), 0.5 M NaCl, 0.5% SDS, 0.1% DTT	5.3

**Table 3:** Description the degree of hydrolysis (DH, %) using alcalase and papain enzymes.

*Cultivar*	DH % Alcalase Digestion	DH % Papain Digestion
*Frantoio*	26.9%	12.5%
*Leccino*	27.5%	15.7%
*Moraiolo*	15.3%	14.7%

**Table 4:** Potential bioactivities according to PeptideRanker and *BIOPEP* database search.

Score ^a^	Sequence	Potential Bioactive Peptides ^b^	Biological Functions ^c^
0.998974	FF	FF	DPP IV inhibitor
0.996643	MF	MF	DPP IV inhibitor
0.994712	GF	GF	DPP IV inhibitor,
0.994084	GGFF	GF, GG, FF	DPP IV inhibitor
		GF	DPP III inhibitor
0.993120	FFJ	FF	DPP IV inhibitor
0.991656	WPM	WP, PM	DPP IV inhibitor
0.989681	VY	VY	DPP IV inhibitor, antioxidant
0.987345	GGF	GF, GG	DPP IV inhibitor
		GF	DPP III inhibitor
0.985719	FR	FR	DPP IV inhibitor
0.977983	WJAF	AF	DPP IV inhibitor
0.977525	FGQW	QW	DPP IV inhibitor
0.974885	WY	WY	DPP IV inhibitor, antioxidant
0.968518	SMF	MF	DPP IV inhibitor
0.962442	FFJA	FF	DPP IV inhibitor
0.954571	FNFJ	NF, FN	DPP IV inhibitor
0.952926	HW	HW	DPP IV inhibitor
0.951176	FN	FN	DPP IV inhibitor
0.948462	FFDR	FF, DR	DPP IV inhibitor
0.947101	WSM	WS	DPP IV inhibitor
0.946135	QF	QF	DPP IV inhibitor
0.941387	AGRF	AG	DPP IV inhibitor
		RF	DPP III inhibitor
0.940972	QGF	GF, QG	DPP IV inhibitor
0.937189	FPAG	PA, FP, AG	DPP IV inhibitor
0.915352	AYF	AY	DPP IV inhibitor
0.913344	DGJF	DG	DPP IV inhibitor, DPP III inhibitor
0.912957	WVAF	AF, VA, WV	DPP IV inhibitor
0.909891	WQ	WQ	DPP IV inhibitor
0.906764	KF	KF	DPP IV inhibitor
0.905338	NGJF	NG	DPP IV inhibitor
0.894996	FJPH	PH	DPP IV inhibitor
0.888582	MPJ	MP	DPP IV inhibitor
0.887708	KGF	GF, KG	DPP IV inhibitor
0.887194	JAF	AF	DPP IV inhibitor
0.881235	AFPA	PA, FP, AF	DPP IV inhibitor
0.879704	AYFG	AY	DPP IV inhibitor, antioxidant
		YF	DPP IV inhibitor
0.874411	SFY	FR	DPP IV inhibitor
		QR	DPP IV inhibitor
0.873986	WSMH	MH, WS	DPP IV inhibitor
0.867049	SFJ	SF	DPP IV inhibitor
0.867026	FNR	FN NR	DPP IV inhibitor
0.866328	PFGD	FG, GD	DPP IV inhibitor
			DPP III inhibitor
0.865974	GPR	GP	DPP IV inhibitor
0.863356	RFN	FN	DPP IV inhibitor
0.85592	HWJY	HW	DPP IV inhibitor
0.849148	MR	MR	DPP IV inhibitor
0.846587	RPFD	RP	DPP IV inhibitor
		PF	DPP IV inhibitor
0.843478	MY	MY	DPP IV inhibitor, antioxidant
0.835091	JSF	SF	DPP IV inhibitor
0.833322	AAF	AF, AA	DPP IV inhibitor
0.825853	FQR	FQ	DPP IV inhibitor
0.820516	SAF	AF	DPP IV inhibitor
0.798127	JWQ	WQ	DPP IV inhibitor
0.796676	WJYN	YN	DPP IV inhibitor
0.785013	YMDM	YM	DPP IV inhibitor
0.768611	GJYP	YP	DPP IV inhibitor
0.761687	RFT	TF	DPP IV inhibitor
			DPP III inhibitor
0.759378	JFQ	FQ	DPP IV inhibitor
0.754757	JJSF	SF	DPP IV inhibitor
0.739381	YP	YP	DPP IV inhibitor
0.728473	FYP	YT	DPP IV inhibitor
0.72244	WVEF	VE	DPP IV inhibitor
		WV	DPP IV inhibitor
0.720745	SRSF	SF	DPP IV inhibitor
0.720069	GFE	GF	DPP IV inhibitor, DPP III inhibitor
0.717504	QQF	QF, QQ	DPP IV inhibitor
0.717158	JSAF	AF	DPP IV inhibitor
0.714532	AQFJ	QF	DPP IV inhibitor
0.713582	AAFJ	AF, AA	DPP IV inhibitor
0.693293	MA	MA	DPP IV inhibitor
0.689222	GQGP	GP, QG	DPP IV inhibitor
0.679365	JVF	VF	DPP IV inhibitor
0.659026	KGFA	GF	DPP IV inhibitor
		KG	DPP IV inhibitor
		FA	DPP IV inhibitor
0.65522	SJVF	VF	DPP IV inhibitor
0.642689	GQP	QP	DPP IV inhibitor
0.64074	JPVM	VM	DPP IV inhibitor
		PV	DPP IV inhibitor
0.636164	HFQ	FQ	DPP IV inhibitor
		HF	DPP IV inhibitor
0.623398	MPJQ	MP	DPP IV inhibitor
0.612254	SFJQ	SF	DPP IV inhibitor
0.601894	KFN	KF	DPP IV inhibitor
		FN	DPP IV inhibitor

^a^ According to PeptideRanker database; http://bioware.ucd.ie/~compass/biowareweb/Server_pages/peptideranker.php, accessed on 4 May 2022. ^b,c^ According to BIOPEP-UWM database; https://biochemia.uwm.edu.pl/biopep-uwm/, accessed on 4 May 2022.

## Data Availability

Data is contained within the article and supplementary material.

## Supplementary Materials

The following supporting information can be downloaded at: https://www.mdpi.com/article/10.3390/antiox11091730/s1, Table S1: Table olive seed peptides SX-SY; Figure S1: Effect of *Frantoio*, *Leccino*, and *Moraiolo* samples hydrolyzed with alcalase and papain on the Caco-2 cells viability by MTT; Figure S2: Effect of PH and AH on the co-culture Caco-2/STC-1 cells viability; Figure S3: Effect of PH and AH on the GLP-1 concentration levels (expressed as pM) in Caco-2-alone cells. References [1,2,3,4,5,6,7,8,9,10] are cited in the supplementary materials.
