# Supplementary material for: Exploitation of Olive (Olea europaea L.) Seed Proteins as Upgraded Source of Bioactive Peptides with Multifunctional Properties: Focus on Antioxidant and Dipeptidyl-Dipeptidase—IV Inhibitory Activities, and Glucagon-like Peptide 1 Improved Modulation
Source: Antioxidants (Basel). 2022 Aug 31;11(9):1730. doi: 10.3390/antiox11091730 (PMC9495363; doi:10.3390/antiox11091730)
Supplement: Supplementary file 1 [file antioxidants-11-01730-s001.zip › antioxidants-1879050-supplementary.pdf]

Supplementary Materials

Exploitation of olive (*Olea europaea L*) seed proteins as upgraded source of bioactive peptides with multifunctional properties: focus on antioxidant and dipeptidyl-dipeptidase– IV inhibitory activities, and GLP-1 improved modulation, and glucagon-like peptide 1 improved modulation.

Martina Bartolomei [1], Anna Laura Capriotti [2], Yuchen Li [3], Carlotta Bollati [1], Jianqiang Li [1], Andrea Cerrato [2], Lorenzo Cecchi [4], Raffaele Pugliese[5], Maria Bellumori [4], Nadia Mulinacci [4], Aldo Laganà [2], Anna Arnoldi[1], Carmen Lammi [1,*]

Department of Pharmaceutical Sciences, University of Milan, 20133 Milan, Italy

[2] Department of Chemistry, Sapienza University of Rome, Piazzale Aldo Moro 5, 00185 Rome, Italy

[3] Longping Biotech Co Ltd., 572000 Sanya, China

[4] Department of Neuroscience, Psychology, Drug and Child Health, Pharmaceutical and Nutraceutical Section, University of Florence, 50019 Florence, Italy

[5] NeMO Lab, ASST Grande Ospedale Metropolitano Niguarda, 20162 Milan, Italy

* Correspondence: carmen.lammi@unimi.it; Tel.: +39-02-50319372

2. Materials and Methods

2.1. Chemical and samples

All chemicals and reagents were of analytical grade and from commercial sources. Tris(hydroxymethyl)aminomethane (Tris), hydrochloric acid (HCl), Sodium chloride (NaCl), 1,4-dithiothreitol (DTT), hexane, Papain and Alcalase enzyme, sodium dodecyl sulfate (SDS), 3-(4,5-dimethylthiazol-2-yl)-2,5-diphenyltetrazolium bromide (MTT), ROS and lipid peroxidantion (MDA) assay kits were from Sigma-Aldrich (St. Louis, MO, USA). Bovine serum albumin (BSA), Bradford reagent, and Coomassie Blue G-250 were purchased from Bio-Rad (Hercules, CA, USA). The DPP-IV enzyme and the substrate solution [5 mM H-Gly-Pro conjugated to aminomethylcoumarin (H-Gly-Pro-AMC)] were provided by Cayman Chemicals (Michigan, USA). Dulbecco's modified Eagle medium (DMEM), fetal bovine serum (FBS), L- glutamine, phosphate buffered saline (PBS), penicillin/streptomycin, 24 and 96- well plates were from Euroclone (Milan, Italy).

2.2. Protein extraction from olive seed

Olive seed were grounded with a domestic mill. Olive seed powder was defatted with hexane for 1h (ratio 1:20 w/v) under magnetic stirring. After drying, the defatted powder was subjected to protein extraction. In details, 1,5 g of defatted powder were mixed with 30 mL of extracting solution containing UREA 6M, 0.1 M Tris-HCl (pH 8), 0.5 M NaCl, 0.5% SDS, 0.1% DTT. The mixture has been stirred for 2h at 4 °C and centrifuged at 4000g for 20 min at 4 °C. The supernatant was collected and dialyzed against 1 M Tris-HCl (pH 8) and stored at -20 °C until use. The protein concentration was determined by the colorimetric Bradford using BSA as a standard curve. The protein extraction protocol was evaluated by SDS- PAGE (12% polyacrylamide gel), with

Tris–glycine buffer (pH 8.3, 0.1% SDS). Staining was performed with Coomassie Blue and destaining with 7% (v/v) acetic acid in water.

2.3. Protein Solubility (PS), Water Binding Capacity (WBC) and Oil Binding Capacity (OBC)

PS was determined according to a method described previously [1] with slight alterations. Each sample 0,2 g was dispersed into 4 mL of 0.1 M phosphate buffer solutions (at pH 2.0 to 12.0), stirred for 20 min. After the pH adjustment the samples were stirred 30 min at RT and then centrifuged at 14,000 rpm for 30 min. The protein concentration in the samples was determined according to the Bradford assay using BSA as a standard. PS was expressed as percentage ratio of supernatant protein content to the total protein content. All determinations were conducted in triplicate. WBC was assessed as previously described [2]. Briefly, 1 g of sample was dispersed in 10 mL distilled water in a 15 mL pre-weighed centrifuge tube. The dispersions were stirred for 30 min and then centrifuged at 7000 g for 25 min at room temperature (RT). The supernatant was discarded, and the tubes was weighed to determine the amount of retained water per gram of sample. The OBC determined according to a method described previously [1] with some slight modifications. In details, 1 g of sample was dispersed in 10 mL sunflower oil and vortexed for 1 min. The mixture was incubated at RT for 30 min and then centrifuged at 7000 g for 25 min at RT. The resulting supernatant was carefully decanted, and the tube containing the precipitation weighed.

2.4. Free Sulfhydryl Group Determination

Ellman's reagent (DTNB) is a compound used for quantifying free sulfhydryl groups in solution observing the production of a yellow-colored product when it reacts with sulfhydryl groups. Briefly, the Ellman's reagent was prepared as follows: 4 mg of DTNB reagent was added to 1 mL of Tris-glycine buffer (0.086 M Tris, 0.09 M glycine, 4 mM EDTA, pH 7.0). Each solution was diluted in Tris-glycine buffer (w/v 0.15%). Then, 5 μL of Ellman's reagent was added to 200 μL of protein suspension. The resulting protein suspensions were incubated at RT for 15 min under shaking and then centrifuged at 10,000 g for 10 min at RT. The absorbance was measured at 412 nm.

2.5. Intrinsic Fluorescence Spectroscopy

The intrinsic fluorescence spectrum of each sample was obtained using a fluorescence spectrophotometer (Synergy H1, Biotek, Bad Friedrichshall, Germany). The samples were diluted in phosphate-buffered saline (PBS, 10 mM, pH 7.0) in order to reach the equal concentration of 0.05 mg/mL and transferred in Greiner UV-Star® 96 well plates flat bottom clear cyclic olefin copolymer (COC) wells (cycloolefine). The excitation wavelength was set as 280 nm, while the excitation and emission slit widths were each set as 5 nm. The emission wavelength range was set up from 300 to 450 nm, and the scanning speed was 10 nm/s.

2.6 Raman Spectroscopy

Raman analysis was conducted using Progeny™ spectrophotometer (Rigaku Corporation, Japan) with a 1,064 nm laser source, and selectable laser output set at 490 mW. The spectral range is 200–2,500 cm^{-1} with transmission-type volume phase grating. The spectral resolution is 15–18 cm^{-1} and the detector is a thermoelectrically cooled indium gallium arsenide (InGaAs). The samples were analyzed with 60 cumulative scans with optimized laser power, aperture size, and duration (7 s) per exposure in order to achieve the best signal to noise ratio. All of the obtained spectra were reported using Origin™ 8 software.

2.7. Olive seed protein hydrolysis for releasing bioactive peptides

The enzymatic hydrolysis of olive seed proteins was performed using Alcalase and Papain enzymes using optimal hydrolysis conditions (Table 1). After incubation, all reactions were blocked by heating at 95 °C for 5 min. The supernatants were taken for subsequent determination of the degree of hydrolysis (% DH). For the kinetics study, 40 μL of each hydrolysis solution was pipetted out for blocking the reaction at 0, 15, 30, 60, 90, 120, 150, 180, 210, 240 min incubation time points. Each hydrolysate was passed through ultrafiltration (UF) membranes with a 3 kDa cut-off, using a Millipore UF system (Millipore, Bedford, MA, USA). All recovered peptides were lyophilized and store -80 °C until use. The %DH for each hydrolysate was identified by the o-phthaldialdehyde (OPA) method as previously described [3].

2.8. Short peptide purification and analysis by high-performance liquid chromatography-high resolution mass spectrometry

Before analysis, short-sized peptides were purified from longer peptides and other macromolecules using a solid-phase extraction cartridge packed with 500 mg of Carbograph 4 with a procedure that was optimized in a previous study [4]. The purified samples were then subject to HPLC-HRMS analysis using a Vanquish binary pump H (Thermo Fisher Scientific) coupled through a heated electrospray (ESI) source to a hybrid quadrupole-Orbitrap mass spectrometer Q Exactive (Thermo Fisher Scientific). The separation of 10 μL of each sample was carried out on a Kinetex XB-C18 (100 × 2.1 mm, 2.6 μm particle size, Phenomenex, Torrance, USA) with chosen flow rate, column temperature, gradient and ESI source parameters as previously reported without any modification [5]. An untargeted suspect screening approach was chosen for the mass spectrometric method, based on the implementation of an inclusion list of m/z derived from all unique masses of short peptides. Data acquisition was performed in the range 150-750 with a resolution of 70,000 (FWHM, m/z 200). HCD fragmentation was performed at 40% normalized collision energy at resolution of 35,000 (FWHM @m/z 200) in top 5 DDA mode. All samples were run in triplicate analysis and raw data files were acquired by Xcalibur software (version 2.2, Thermo Fisher Scientific). Short peptide identification was aided by the Compound Discoverer software (v 3.1, Thermo Fisher Scientific) with a dedicated data processing workflow that was based on the use of a local mass list with IDs, molecular weights, and molecular formulas of all the combination of the 20 natural amino acids in short-sized peptide (the same list was also employed as an inclusion list in the mass spectrometric method) [6]. Raw data files of each sample and a blank sample were simultaneously processed for peak alignment and remotion of all compounds that were present in the blank sample, that were not associated to one of the IDs in the mass list, or that were not associated to an MS/MS spectrum. Manual validation of the short peptide sequences was aided by mMass 5.5, that allows generating *in-silico* MS/MS spectra [7].

2.9. Profile of Potential Biological Activities and Peptide Ranking

The open access tool PeptideRanker (http://bioware.ucd.ie/~compass/biowareweb/Server_pages/peptideranker.php), a web-based tool used to predict the eventuality of biological activity of peptide sequences, was used to forecast the potential bioactivities of olive seed peptides [1]. Using *N*-to-1 neural network probability, PeptideRanker provides peptide scores in the range of 0-1. The peptides with a score higher than 0.6 were considered as potentially "bioactive". Subsequently, the lists of best-ranked peptides were submitted to the web-available database BIOPEP (http://www.uwm.edu.pl/biochemia/index.php/pl/biopep/).

2.10. Cell Culture

Caco-2 cells, obtained from INSERM (Paris, France) and STC-1, bought from ATCC (HB- 8065, ATCC from LGC Standards, Milan, Italy) were routinely sub-cultured following a previously optimized protocol [9] and maintained at 37°C in a 90% air/10% CO^2 atmosphere in DMEM containing 25 mM of glucose, 3.7 g/L of $NaHCO_3$, 4 mM of stable L-glutamine, 1% non-essential amino acids, 100 U/L of penicillin and 100 μg/L of streptomycin (complete medium), supplemented with 10% heat-inactivated FBS. For the co-culture, the STC-1 and Caco-2 cells were cultured in a 1:5 ratio for 48h before proceeding with treatments.

2.11. 3-(4,5-Dimethylthiazol-2-yl)-2,5-Diphenyltetrazolium Bromide (MTT) Assay

A total of 3 x 10^4 Caco-2 cells/well were seeded in 96-well plates and treated with 0.1, 0.5, 1.5, and 5 mg mL^{-1} of hydrolysates and/or vehicle (H_2O) in complete growth medium for 48 h at 37 °C under a 5% CO^2 atmosphere, following the procedure previously reported [10]. For the co-culture system a total of 3 x 10^4 Caco-2 cells-STC-1 /well cells were seeded in 96-well plates and treated with 0.1, 0.5, and 5 mg mL^{-1} of hydrolysates and/or vehicle (H_2O) following the same conditions described above.

2.12. Antioxidant activity of olive seed hydrolysates

2.12.1. Diphenyl-2-picrylhydrazyl radical (DPPH) assay

The DPPH assay was performed by a standard method with a slight modification. Briefly, 45 μL of 0.0125 mM DPPH solution (dissolved in methanol) was added to 15 μL of the OA and OP hydrolysates at the final concentrations of 1.0, 2.5 and 5.0 mg/mL. The reaction for scavenging the DPPH radicals was performed in the dark at room temperature and the absorbance was measured at 520 nm after 30 min incubation.

2.12.2. 2,2′ -Azino-bis(3-ethylbenzothiazoline-6-sulfonic) acid diammonium salt assay

The Trolox equivalent antioxidant capacity (TEAC) assay is based on the reduction of the 2,2-azino-bis-(3-ethylbenzothiazoline-6-sulfonic) acid (ABTS) radical induced by antioxidants. The ABTS radical cation (ABTS+•) was prepared by mixing a 7 mM ABTS solution (Sigma- Aldrich, Milan, Italy) with 2.45 mM potassium persulfate (1:1) and stored for 16 h at room temperature and in dark. To prepare the ABTS reagent, the ABTS+• was diluted in 5 mM phosphate buffer (pH 7.4) to obtain a stable absorbance of 0.700 (±0.02) at 730 nm. For the assay, 10 μL of OA and OP hydrolysates at the final concentrations of 0.01, 0.05 and 1 mg/mL were added to 140 μL of diluted the ABTS+•. The microplate was incubated for 30 min at 30 ◦C and the absorbance was read at 730 nm using a microplate reader Synergy H1 (Biotek). The TEAC values were calculated using a Trolox (Sigma-Aldrich, Milan, Italy) calibration curve (60-320 μM).

2.12.3. FRAP assay

The FRAP assay evaluates the ability of a sample to reduce ferric ion (Fe3+) into ferrous ion (Fe2+). Thus, 10 μL of OA and OP hydrolysates at the final concentrations of 0.01, 0.05 and 1 mg/mL were mixed with 140 μL of FRAP reagent. The FRAP reagent was prepared by mixing 1.3 mL of a 10 mM TPTZ (Sigma-Aldrich, Milan, Italy) solution in 40 mM HCl, 1.3 mL of 20 mM $FeCl_3 \times 6 H_2O$ and 13 mL of 0.3 M acetate buffer (pH 3.6). The microplate was incubated for 30 min at 37°C and the absorbance was read at 595 nm. Absorbances were recorded on a microplate reader Synergy H1 (Biotek).

2.12.4. Fluorometric intracellular ROS assay

For cells preparation, 3×10^4 Caco-2 cells/well were seeded on a black 96-well plate overnight in growth medium. The day after, the medium was removed, 50 μL/well of Master Reaction Mix was added and the cells were incubated at 5% CO_2, 37 °C for 1 h in the dark. Then, the PH and AH hydrolysates were added to reach the final concentrations of 0.1 mg/mL and incubated at 37 ◦C for 24 h. To induce ROS, cells were treated with H_2O_2 at a final concentration of 1.0 mM for 30 min a 37 °C in the dark and fluorescence signals (ex./em. 490/525 nm) were recorded using a microplate reader Synergy H1 (Biotek).

2.12.5. Lipid peroxidation (MDA) assay

Caco-2 cells (2.5×10^5 cells/well) were seeded in a 24 well plate and, the following day, they were treated the PH and AH hydrolysates for 24 h at 37 °C under 5% CO_2 atmosphere. The day after, cells were incubated with H_2O_2 1 mM or vehicle (H_2O) for 1 h, then collected and homogenized in 150 μL ice-cold MDA lysis buffer containing 3 μL of butylated hydroxytoluene (BHT). Samples were centrifuged at 13,000 g for 10 min, then were filtered through a 0.2 μm filter to remove insoluble material. To form the MDA-TBA adduct, 300 μL of the TBA solution were added into each vial containing 100 μL samples and incubated at 95 ◦C for 60 min, then cooled to RT for 10 min in an ice bath. For analysis, 100 μL of each reaction mixture were pipetted into a clear 96 well plate and the absorbance were measured at 532 nm using the microplate reader Synergy H1 (Biotek). To normalize the data, total proteins for each sample were quantified by Bradford method.

2.13 Antidiabetic activity of olive seed hydrolysates

2.13.1. In vitro measurement of the DPP-IV inhibitory activity

The experiments were carried out in triplicate in a half volume 96 well solid plate (white) using conditions previously optimized [8]. A total of 50.0 μL of each reaction was prepared in a microcentrifuge tube adding 30.0 μL of 1 × assay buffer [20mM Tris-HCl, pH 8.0, containing 100 mM NaCl, and 1 mM EDTA], 10.0 μL of each sample (at the final concentration of 0.1, 0.5, 1.0, and 1.5 mg/mL), sitagliptin at 1.0 μM (positive control) and 10.0 μL of purified human recombinant DPP-IV enzyme. Next, reagents were transferred in each well of the plate and each reaction was started by adding 50.0 μL of substrate solution (5mM H-Gly-Pro-AMC) and incubated at 37 °C for 30 min. Fluorescence signals were measured using the Synergy H1 fluorescent plate reader from Biotek (excitation/emission wavelength 360/465 nm).

2.13.2. Evaluation of the Inhibitory Effect of olive seed hydrolysates on Cellular DPP-IV Activity

A total of 3×10^4 Caco-2 cells/well were seeded in black 96-well plates with clear bottom. The second day after seeding, the spent medium was discarded and cells were treated with 100 μL/well of OA and OP at the concentration of 1.5 and 5 mg/mL or vehicle (C) in growth medium for 3 h at 37°C. Afterwards, treatments were removed and Caco-2 cells were washed once with 100 μL of PBS without Ca^{2+} and Mg^{2+}, before the

addition to each well of 100 μL of Gly-Pro-AMC substrate at the concentration of 50.0 μM in PBS without Ca^{2+} and Mg^{2+}. Fluorescence signals deriving from the release of free AMC were measured using a Synergy H1 fluorescence microplate reader from BioTek (excitation/emission wavelength 350/465 nm respectively) every 1 min for up to 10 minutes.

2.13.3. Evaluation of the GLP-1 stability and secretion at cellular level

STC-1 GLP-1 secretion was measured by an active GLP-1 ELISA kit (catalog no. EGLP-35K; Millipore, Watford, UK). In details, a total of 2.4 x 10^4 Caco-2 cells and 6 x 10^3 STC-1 cells/well or 6 x 10^3 STC-1 cells/well were seeded in 96-well plates. After 48h cells were treated with Sitagliptin (1 μM), PH and AH (5 mg/mL) or vehicle (C) in growth medium for 1h. After the treatment, the supernatant was collected and centrifugated at 500 G, 4°C for 5 min and incubated in 96-well microplates coated with a monoclonal antibody for overnight (20 to 24 hours) at °4C. After washing the wells, the detection conjugated was added for 2h. The wells were washed, and then the substrate solution was added for 20 min. The reaction was stopped by a stop solution, and then the plate was read to with an excitation/emission wavelength of 355 nm/460 nm with a Synergy H1 microplate reader (Biotek Instruments, Winooski, VT, USA).

2.14. Statically Analysis

All measurements were performed in triplicate and results were expressed as the mean ± standard deviation (s.d.), where p-values < 0.05 were considered to be significant. Statistical analyses were performed by ONE and 2way ANOVA followed by Dunnett's and Tukey's post-test (Graphpad Prism 9, GraphPad Software, La Jolla, CA, USA).

3. Results

Effect of the olive seed protein hydrolysates on cell vitality

MTT experiments were conducted for sorting out those concentrations of the hydrolysates that may potentially determine cytotoxic effects on Caco-2 cells. Notably, no cytotoxic effect was observed up to 5.0 mg/mL, after 48 h treatment.

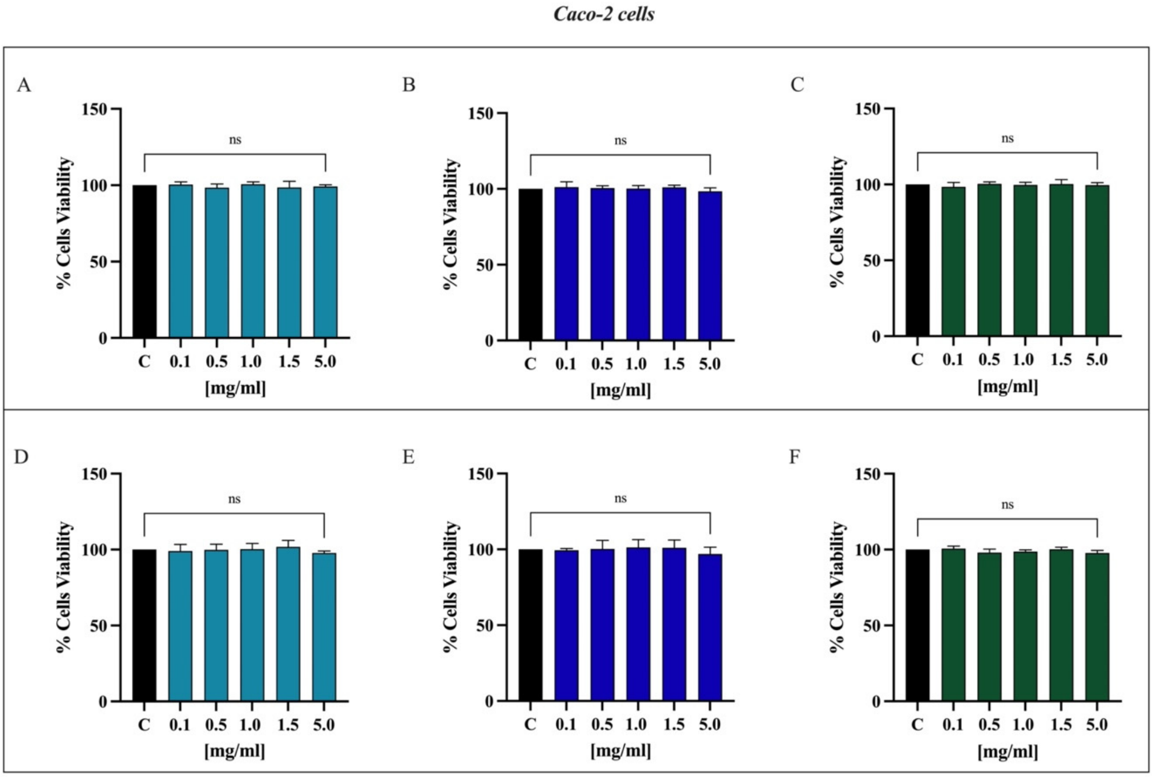

Figure S1. MTT assay. Effect of *Frantoio* (a, d), *Leccino* (b, e) and *Moraiolo* (c, f), samples hydrolyzed with Alcalase (top) and Papain (bottom) on the Caco-2 cells viability. Data represent the mean ± s.d. of three independent experiments performed in triplicate. ns: not significant.

MTT experiments were conducted also on the co-culture system to determine potentially cytotoxic effects. Notably, no cytotoxic effect was observed up to 5.0 mg/mL, after 48 h treatment.

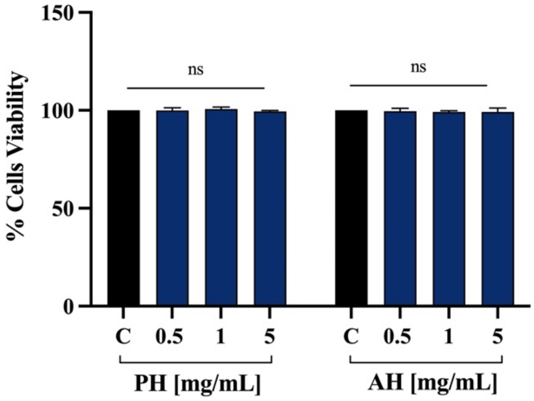

Figure S2. MTT assay. Effect of PH and AH on the co-culture Caco-2/STC-1 cells viability. Data represent the mean ± s.d. of three independent experiments performed in triplicate. ns: not significant.

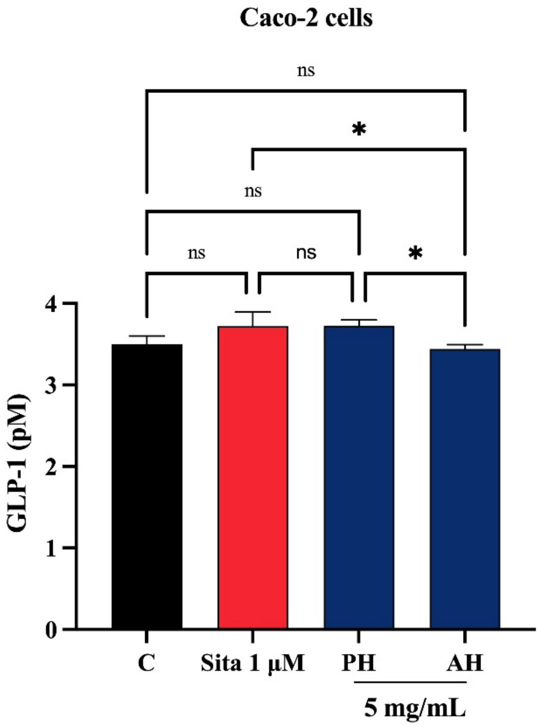

Figure S3. Effect of PH and AH on the GLP-1 concentration levels (expressed as pM) in Caco-2-alone cells. Data points represent averages ± s.d. of three independent experiments in duplicate. All data sets were analyzed by one-way ANOVA followed by Tukey's post-hoc test. C: control sample; Sita: Sitagliptin; ns: not significant (*) $p < 0.005$ vs. control (C).
